# Brain capillary endothelial-like cells show altered barrier functionality and reduced transport of amyloid β in late-onset Alzheimer disease

**DOI:** 10.1186/s12987-025-00753-7

**Published:** 2026-01-22

**Authors:** Carla Hartmann, Undine Haferkamp, Antje Appelt-Menzel, Janica Barenberg, Andreas Brachner, Toni Ehrhard, Julia Feldhaus, Anna Gerhartl, Thomas Hollemann, Linda Anna Michelle Kulka, Selin Leckzik, Jennifer Leu, Marcel Seungsu Woo, Manuel Alexander Friese, Alzheimer’s Disease Neuroimaging Initiative, Marco Metzger, Winfried Neuhaus, Sabrina Oerter, Heidi Olzscha, Andreas Pich, Dagmar Riemann, Ole Pless, Dan Rujescu, Matthias Jung

**Affiliations:** 1 Institute for Physiological Chemistry (IPC), Medical Faculty of the Martin Luther University Halle-Wittenberg, 06114 Halle (Saale), Germany; 2 Fraunhofer Institute for Translational Medicine and Pharmacology ITMP, Discovery Research Screening Port, 22525 Hamburg, Germany; 3 Fraunhofer Institute for Silicate Research ISC, Translational Center Regenerative Therapies (TLC-RT), 97070 Würzburg, Germany; 4 Chair Tissue Engineering and Regenerative Medicine (TERM), University Hospital Würzburg, 97070 Würzburg, Germany; 5 Center Health and Bioresources, Competence Unit Molecular Diagnostics, AIT Austrian Institute of Technology GmbH, Vienna, 1210 Austria; 6 Department of Medicine, Faculty of Medicine and Dentistry, Danube Private University, Krems, 3500 Austria; 7 Medical School Hamburg MSH, Institute of Molecular Medicine, University of Applied Sciences and Medical University, 20457 Hamburg, Germany; 8 Institute of Toxicology, Hannover Medical School, 30625 Hannover, Germany; 9 Department Medical Immunology, Martin Luther University Halle-Wittenberg, 06118 Halle (Saale), Germany; 10 Department of Psychiatry and Psychotherapy, Division of General Psychiatry, Medical University of Vienna, Vienna, 1090 Austria; 11 Translational Neurodegeneration Laboratory, Department of Neurology, University Medical Center Hamburg-Eppendorf, 20251 Hamburg, Germany; 12 Institute of Neuroimmunology and Multiple Sclerosis, University Medical Center Hamburg-Eppendorf, 20251 Hamburg, Germany; 13 Translational Neuroimaging Laboratory, McConnell Brain Imaging Center, Montréal Neurological Institute, McGill University, Montréal, 3801 Canada; 14 Department of Neurology and Neurosurgery, Montréal Neurological Institute, McGill University, Montréal, 3801 Canada; 15 Department of Neurology, University Medicine Halle (UMH), 06120 Halle (Saale), Germany

**Keywords:** Apolipoprotein E, Human induced pluripotent stem cells, Blood-brain barrier, Amyloid beta, Brain capillary endothelial-like cells, Late-onset Alzheimer disease

## Abstract

**Background:**

With the progression of late-onset Alzheimer disease (LOAD), there is a dysregulation and then a breakdown of the blood-brain barrier (BBB). An important pathological feature in the brains of patients is the accumulation of amyloid beta (Aβ) peptides. Their aggregation leads to the formation of particularly harmful Aβ oligomers (Aβ-O). Unfortunately, our understanding of changes in the blood-brain barrier, particularly with regard to the effects of Aβ-O, is still very limited.

**Methods:**

This study investigated a LOAD-specific and induced pluripotent stem cell (hiPSC)-based in vitro model of the BBB for disease mechanisms and validated the findings in two independent laboratories. This study also investigated Aβ transport across the BBB. Furthermore, obtained in vitro findings were confirmed in the cerebrospinal fluid proteome of a LOAD patient cohort.

**Results:**

Control and LOAD hiPSCs exhibited comparable efficiency in forming brain capillary endothelial-like cells (BCECs). Although transendothelial electrical resistance (TEER) assessments indicated no significant differences in barrier tightness between LOAD and control BCECs, high-throughput multiplex qPCR analysis revealed subtle alterations in barrier integrity. This included changes in various barrier markers, such as mucins (MUC1, MUC20), aquaporins (AQP5, AQP10), junctional transcripts (CLDNs, TJP1, OCLN), and receptors (LRP1, INSR, LSR), which were confirmed in LOAD patients. High-content imaging and flow cytometry indicated reduced cadherin 5 (CDH5) levels in LOAD BCECs. Importantly, the results also highlighted a difference in the transport of Aβ-O across the BBB.

**Conclusion:**

This model demonstrates a LOAD-relevant phenotype with decreased Aβ transport and alterations in key transcripts and could thus serve for future translational studies to rescue pathogenic phenotypes.

**Graphical Abstract:**

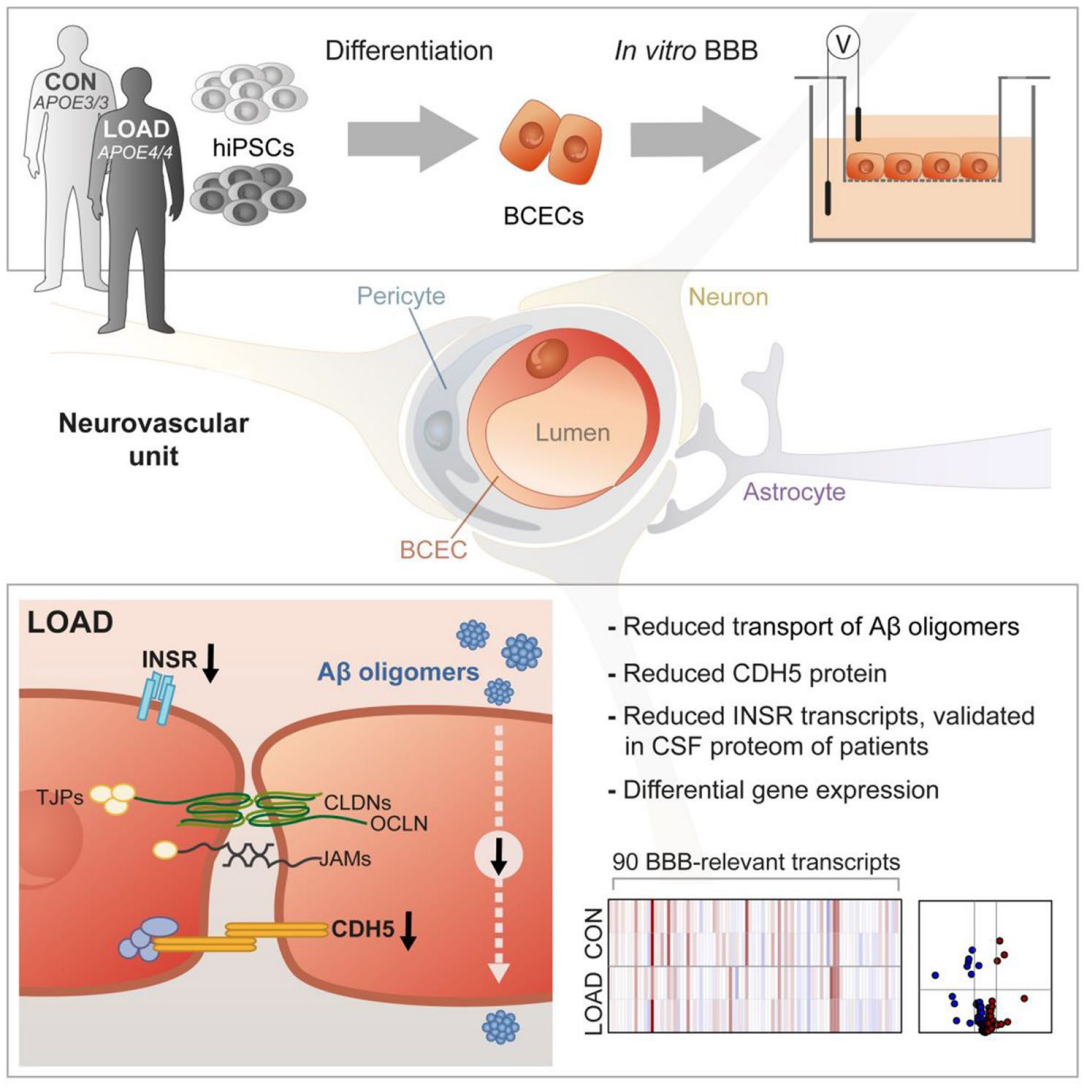

**Supplementary Information:**

The online version contains supplementary material available at 10.1186/s12987-025-00753-7.

## Background

In 2021, the World Health Organization found that late-onset Alzheimer disease (LOAD) and other forms of dementia are the seventh leading cause of death worldwide [1]. The number of people affected is expected to continue to rise. The pathophysiology of LOAD is poorly understood, which has hampered the development of preventive and curative treatments [2]. LOAD is associated with the formation of neurotoxic extracellular plaques consisting of misfolded amyloid beta (Aβ) peptides, which form particularly harmful Aβ oligomers (Aβ-O), as well as with the accumulation of intracellular neurofibrillary tangles consisting of hyperphosphorylated tau proteins (tau pathology). This is associated with the loss of vascular function of the neurovascular unit (NVU), which consists of specialised brain capillary endothelial cells and other central nervous system (CNS) cells [3–5]. The NVU is responsible for the degradation and removal of neurotoxic substances from the CNS [6].

The blood-brain barrier (BBB) is anatomically a part of the NVU and regulates the exchange of substances between the blood circulation and brain parenchyma, protecting against harmful agents while supplying the brain with essential nutrients [7]. In this process, brain capillary endothelial cells have a central role in forming a physical barrier to prevent paracellular transport [8]. Tight junctions (TJs) between brain capillary endothelial cells seal the intercellular space and restrict the unregulated, paracellular transport of mainly hydrophilic compounds. Members of the ATP-binding cassette (ABC) and solute carrier (SLC) transporter protein families regulate the transcellular transport of small molecules. Thus, the transport and metabolic barrier functions of brain capillary endothelial cells significantly contribute to maintaining brain homeostasis. The NVU can undergo changes over time that can contribute to the development of age-related diseases, including LOAD [9].

Several studies indicate that Aβ triggers a pathogenic cascade that mediates neurovascular and neuronal dysfunction in LOAD [10–12]. During Aβ clearance through the glymphatic or lymphatic pathways, three different degradation mechanisms have been postulated. Aβ might be degraded in the extracellular space by various proteases [13]. Cells in the NVU can endocytose Aβ and remove it by lysosomal degradation [14]. Endothelial cells likely mediate Aβ transport across the BBB in large part by expressing various receptors and transporters, such as low-density lipoprotein receptor-related protein 1 (LRP1), ATP-binding cassette subfamily B member 1 (ABCB1, also known as MDR1 or PG-P), and the receptor for advanced glycation end products (AGER). Aβ injected into the mouse hippocampus is cleared less efficiently by interstitial fluid drainage with age, likely due to thickening of the vascular basement membrane with altered extracellular matrix components [15].

Animal models have been used to study the NVU in the context of LOAD; however, there are limitations regarding their ability to model LOAD due to species-specific differences in brain anatomy and physiology [16–18]. Only very old mice show signs of neurodegeneration, which is then limited to very specific regions of the brain. Furthermore, only transgenic mice with mutated tau protein develop neurofibrillary tangles (tau pathology) [19]. This suggests that even when they show a strong amyloid pathology, the related pathways exhibit differences due to species-specific alterations. Cell lines (such as SH-SY5Y) as well as primary cells (such as hippocampal cells) of human and animal origin have been used in numerous studies investigating LOAD, and some of them also gathered evidence that Aβ clearance is important in LOAD [20, 21]. In addition, other limitations restrict the transferability of the results to the patient. Cell lines often carry highly mutated genomes, while primary cells are scarce and show high batch-dependent variability [22]. Even human cell lines cannot adequately mimic cell physiology and may lack important properties of mature and ageing cells that are required for the onset, progression, and treatment of LOAD [16–18]. These limitations have contributed to the lack of success in developing highly efficacious drugs that target LOAD. Human induced pluripotent stem cells (hiPSCs) are expected to overcome species differences, exhibit lower batch variability, and provide age-dependent cellular phenotypes.

A common problem with many cell culture models is their transferability to different laboratories. On the one hand, this is due to the complexity of the techniques; on the other hand, to their lack of reproducibility. However, easy transferability is crucial for the dissemination of a model for use in the development of therapies. There are already a few studies on the widely used Caco-2 cell line and hiPSCs, among others, which have investigated transferability [23, 24].

To address these current challenges, we recently developed an endothelial in vitro model that can mimic the functional properties of the BBB. For this purpose, we used hiPSCs from LOAD patients with the AD risk variants APOE4/4 and from older adult control subjects with the APOE3/3 haplotype [25–27]. In the present study, we used the endothelial in vitro model that mimics the BBB to investigate Aβ clearance. For a targeted analysis of Aβ oligomer (Aβ-O) transport across hiPSC-derived brain capillary endothelial-like cells (BCECs), we utilised a custom high-throughput qPCR array [28], aiming to interrogate the integrity and functionality of BCECs from different backgrounds. This study revealed significant changes that may contribute to disturbed Aβ transport, rather than BBB leakage, underlying the pathological feature of reduced Aβ clearance in LOAD.

## Methods

### Origin and characterisation of hiPSCs

To generate BCECs, we used hiPSCs derived from a LOAD patient (age 76) who was recruited according to NINCDS-ADRDA criteria [29] at the outpatient clinic of the Department of Psychiatry, University of Munich, Germany. The patient (at age 73) was diagnosed with mild-grade dementia (ICD-10: F00.1) according to the Diagnostic and Statistical Manual of Mental Disorders (DSM-IV) and carried a homozygous APOE ε4 genotype (APOE4). Cognitive tests included the Mini-Mental State Examination (MMSE) and the Consortium to Establish a Registry for Alzheimer Disease (CERAD) neuropsychological assessment battery, including a vocabulary test. An older adult (age 64) was selected as a matched control donor. There was an absence of central neurological disease and psychotic disorders in the control subject, including first-degree relatives, evaluated using the Structured Clinical Interview for DSM-IV (SCID I and II) [30] and the Family History Assessment Module (FHAM) [31]. Cognitive tests included the MMSE and a vocabulary test. The healthy control individual was matched for ancestry (German), gender, and age and carried a homozygous APOE ε3 genotype (APOE3/3). For the generation of hiPSCs, subjects’ blood was collected, and peripheral blood mononuclear cells (PBMCs) were isolated for immortalisation by Epstein-Barr virus infection. The B-lymphoblastoid cell lines (B-LCLs) obtained were then used to generate hiPSCs by electroporation with episomal vectors, as recently described [27]. The somatic donor cells and resulting hiPSCs (MLUi007-J and MLUi009-A) were extensively characterised, the latter for their pluripotency characteristics, differentiation capacity, and genomic integrity (https://hpscreg.eu). The reference hiPSC line iPS(IMR90)-4 (alias WISCi004-B; RRID: CVCL_C437) was obtained from the Wisconsin International Cell Bank (WiCell Research Institute, USA). Another reference hiPSC line, ZIPi013-B (RRID: CVCL_UF44), was established by our laboratory [32].

### hiPSC culture

hiPSCs were grown in mTeSR^TM^1 medium (Stemcell Technologies) supplemented with 1.0% gentamycin (Thermo Fisher Scientific) in adherent feeder-free culture on Matrigel^TM^-coated 6-well plates (VWR, 0.083 mg/ml, 1 ml/well). Matrigel was diluted in Knockout™ Dulbecco’s Modified Eagle Medium (DMEM; Thermo Fisher Scientific). hiPSCs were cultured at 37 °C in a humidified environment with 5% CO_2_ or 5% O_2_, 5% CO_2_, and 90% N_2_ (hypoxia). For enzymatic passaging, cells were treated with 1 mg/ml collagenase IV (Thermo Fisher Scientific) in Knockout™ DMEM for about 30 min at 37 °C or Gentle Cell Dissociation Reagent (Stemcell Technologies) for 7 min at room temperature, followed by rinsing once in Knockout™ DMEM, followed by mechanical dissociation and seeding at a 1:10 − 1:100 split ratio with daily medium replacement.

### Generation of neurons

For the generation of neurons, this study used the reference hiPSC line ZIPi013-B following a previously described protocol [33, 34]. First, hiPSCs were converted into neural progenitor cells (NPCs) expressing paired box 6 (PAX6), SRY-box transcription factor 1 (SOX1), SOX2, nestin (NES) and dachshund family transcription factor 1 (DACH1) using the STEMdiff™ SMADi Neural Induction Kit (Stemcell Technologies). Therefore, hiPSCs were seeded in STEMdiff SMADi Neural Induction Medium (SMADi NIM) supplemented with 10 µM Y-27,632 on AggreWell^TM^800 plates (Stemcell Technologies) at a density of 3 × 10^6^ cells/well. On day 6, embryoid bodies were harvested and cultured on Matrigel-coated 6-well plates in SMADi NIM. On day 12, neural rosettes were manually picked, cultured separately for another four days and then dissociated with Accutase™ (Thermo Fisher Scientific) to obtain NPC monolayer cultures, which were maintained for several passages in STEMdiff Neural Progenitor Medium (NPC medium, Stemcell Technologies) on Matrigel-coated plates for cryopreservation in Bambanker™ (NIPPON Genetics) or for further differentiation into neurons.

For neuronal differentiation, NPCs were seeded at a density of 5 × 10^4^ cells/cm^2^ in NPC medium on poly-L-ornithine/laminin-coated plates (both Thermo Fisher Scientific). The next day, the medium was switched to neural differentiation medium containing Neurobasal™ Plus Medium, 1x B-27™ Plus Supplement, 1× MEM non-essential amino acids (all Thermo Fisher Scientific), 1 µg/ml laminin (Merck), 1x N2 Supplement-A, 1 µM dibutyryl-cAMP, 10 ng/ml L-ascorbic acid, 10 ng/ml brain-derived neurotrophic factor (BDNF), and 10 ng/ml Glial cell line-derived neurotrophic factor (GDNF) (all Stemcell Technologies) supplemented with 5 µM cyclopamine (Stemcell Technologies) for the first week and with 2 µM cytarabine (Merck) for the second week of neuronal differentiation. Thereafter, cells were dissociated with Accutase and cultured on poly-L-ornithine/laminin-coated plates for an additional two weeks until they were used for neurotoxicity assessment of Aβ-O.

### Generation of BCECs

BCECs were generated from hiPSCs as recently described [26]. On day 3, hiPSCs were passaged with Accutase and seeded in mTeSR1 supplemented with 10 µM HA100 (Santa Cruz Biotechnologies) or Y-27632 (Stemcell Technologies) on Matrigel-coated 6-well plates using an established number of cells (7.5–12.5 × 10^3^ cells/cm^2^). On day 0, cells were treated with unconditioned medium containing 78.5% DMEM/F12 without glutamine, 20% Knockout serum replacement, 1 mM GlutaMAX™ or L-glutamine, 1% non-essential amino acids (all Thermo Fisher Scientific), and 0.1 mM β-mercaptoethanol (Merck) to initiate differentiation. On day 6, medium was changed to endothelial cell medium containing 99.5% human endothelial serum-free medium and 0.5% B-27 (all Thermo Fisher Scientific) supplemented with 20 ng/ml FGF2-basic (also known as FGF2; Peprotech) and 10 µM retinoic acid (Stemcell Technologies). On day 8 of differentiation, 1 × 10^6^ cells/cm^2^ were seeded onto collagen IV/fibronectin-coated inserts (0.4-µm-pore size, transparent, 24-well format; Greiner Bio-One), 1 mg collagen IV, and 0.5 mg/ml fibronectin (both Merck) in 1 ml deionised water. On days 9 and 10, cells were cultured in endothelial cell medium without additives.

### Human cohort

All data were retrieved from the Alzheimer’s disease neuroimaging initiative (ADNI) database (adni.loni.usc.edu). The ADNI was launched in 2003 as a public-private partnership, led by Principal Investigator Michael W. Weiner, MD. The primary goal of ADNI has been to test whether serial magnetic resonance imaging (MRI), positron emission tomography (PET), other biological markers, and clinical and neuropsychological assessment can be combined to measure the progression of Mild Cognitive Impairment (MCI) and Alzheimer Disease Dementia (ADD). For up-to-date information, see www.adni-info.org. The study was approved by the Institutional Review Boards of all participating institutions, and written informed consent was obtained from all participants. The data used for the analyses presented herein were accessed on May 6th, 2025. We only included individuals older than 65 years, people without any amyloid or tau pathology, and people with only amyloid pathology. A neocortical [18 F] Florbetapir standardised uptake value ratio (SUVR) > 1.1 [35] or cerebrospinal fluid (CSF) Aβ(1–42) < 981 [36] were used as cut-offs to determine Aβ positivity. Heterozygous and homozygous APOE4 carriers were summarised as APOE4 carriers in our statistical analyses. Our cohort encompassed 148 APOE3 carriers with no Aβ or tau pathology and 51 APOE4 carriers with Aβ deposits in the brain.

### Cerebrospinal fluid analysis

The protein levels and methods can be retrieved from ADNI. Briefly, protein levels were quantified using SomaLogic’s aptamer-based SomaScan 7k, which offers a multiplexed DNA aptamer assay for protein quantification. The data contain the quantitative levels of 7293 aptamers measured in relative fluorescence units (RFU). The methods have been described in detail elsewhere [37]. Parametric t-tests were used for the statistical comparisons.

### Transendothelial electrical resistance (TEER) measurement

TEER values (Ω*cm^2^) were determined as recently described [26]. The resistance value (Ω) was measured using the Millicell ERS-2 (Millipore) and electrode type STX01 (World Precision Instruments). TEER values were determined on days 9 and 10, and also on day 11 for Aβ-O transport studies only. Measurements were performed for at least three independent BCEC differentiations (mean ± SEM). The results were statistically analysed as indicated in the respective figures. BCECs with TEER values higher than 800 Ω*cm^2^ on day 10 were included in this study. This threshold for monocultures corresponds to the maximum values for primary cultures, is usually exceeded by hiSPC-derived cells, and is suitable for the analysis of small molecules [38].

### Sodium fluorescein (NaF) transport assay

NaF permeability (PC_NaF_) was determined by a NaF transport assay as recently described [26]. NaF was determined using a fluorescence reader (Tecan Infinite M1000 Pro, Tecan Infinite 200 PRO) with the following settings: excitation 490 nm and emission 525 nm. All measurements were performed in triplicate and for at least three independent biological assays (mean ± SEM). The results were statistically analysed as indicated in the respective figures.

### High-throughput multiplex qPCR of barrier genes

BCECs were harvested after performing TEER measurements and the NaF transport assay. 90 mRNA transcripts important for BBB integrity and functionality, as well as four internal controls (Additional file 1: Table S1), were evaluated as recently described [26]. The extreme studentized deviate method (Grubbs’ test) was applied to threshold cycle (Ct) values to detect outliers. Afterwards, Ct values were normalised to the geometric mean of four endogenous housekeeping genes (peptidylprolyl isomerase A, PPIA; actin beta, ACTB; glyceraldehyde-3-phosphate dehydrogenase, GAPDH; and beta-2-microglobulin, B2M) and relative quantification was calculated according to the comparative 2^−ΔΔCt^ method [39]. Statistical significance (*p* ≥ 0.05) was performed with Student’s t-test using normalised Ct values.

### Flow cytometry

Cells were washed in phosphate-buffered saline (PBS) and then harvested with Accutase for about 5 min at 37 °C to obtain a single-cell suspension. Next, the reaction was stopped, cells were washed in PBS, and the number of cells was determined using a NucleoCounter™ NC-200 (ChemoMetec). Next, 1 × 10^6^ cells were permeabilised and fixed with a FoxP3 staining buffer set (Miltenyi Biotec, #130-093-142) as recently described [27]. The obtained cell suspension was incubated on ice for 30 min using the following fluorescence-labelled primary antibodies and isotype controls: Human IgG_1_ anti-human cadherin 5 (CDH5, also known as VE-cadherin or CD144), allophycocyanin (APC) (1:10 #130-100-709, Miltenyi Biotec), Human IgG_1_ isotype control APC (1:10, #130-104-615, Miltenyi Biotec), Mouse IgG_1_ anti-human solute carrier family 2 member 1 (SLC2A1, also known as GLUT1), Phycoerythrin (PE) (1:10, #FAB1418P, R&D), Mouse IgG_2b_ isotype control PE (1:10, #IC0041P, R&D), Mouse IgG_1_ anti-human tight junction protein 1 (TJP1, also known as ZO1), fluorescein isothiocyanate (FITC) (1:200, #ab150266, Abcam), and Mouse IgG_1_ isotype control FITC (1:200, #ab91356, Abcam). Finally, cells were washed in permeabilisation buffer and resuspended in FC buffer for analysis. Cells were analysed using the BD FACSCanto™ flow cytometer (BD Biosciences) in three independent experiments (mean ± SEM). The results were statistically analysed as indicated in the respective figures.

### Immunofluorescence analysis

Cells were fixed for 15 min at room temperature using 4% paraformaldehyde and washed three times with 1x PBS. Fixed cells were incubated with 0.1% Triton™ X-100 (Carl Roth) for 15 min or with a combination of 0.1% Triton X-100 and 1% serum (Thermo Fisher Scientific) for 30 min, and stained as recently described [26] using the following primary antibodies: Goat IgG anti-human POU class 5 homeobox 1 (POU5F1, also known as OCT4) (1:500, #sc-8629, Santa Cruz Biotechnology), Mouse IgG_1_ anti-human SOX2 (1:300, #sc-365823, Santa Cruz Biotechnology), Rabbit IgG anti-human MYC proto-oncogene (MYC) (1:100, #sc-764, Santa Cruz Biotechnology), Rabbit IgG anti-human Nanog Homeobox (NANOG) (1:500, #ab109250, Abcam), Rabbit IgG anti-human lin-28 homolog A (LIN28A) (1:500, #11724-1-AP, Proteintech), Mouse IgG_2a_ anti-human SLC2A1/GLUT1 (1:200, #ab40084, Abcam), Rabbit IgG anti-human SLC2A1/GLUT1 (1:200, #ab115730, Abcam), Mouse IgG_1_ anti-human occludin (OCLN) (1:200, #33-1500, Thermo Fisher Scientific), Rabbit IgG anti-human TJP1/ZO1 (1:400 or 1:100, #21773-1-AP, Proteintech), Rabbit IgG anti-human claudin 5 (CLDN5) (1:100, #ab15106, Abcam), and Mouse IgG_1_ anti-human CDH5/VE-cadherin (1:100, #sc-9989, Santa Cruz Biotechnology). Fluorescence-labelled antibodies were used for labelling (1:500, Alexa Fluor™ 488/555/633/647, Thermo Fisher Scientific or 1:400, cyanine 3 (Cy3), Jackson ImmunoResearch). Hoechst 33,342 (Thermo Fisher Scientific) was used for the visualization of the cell nuclei. Fluorescent images were taken using the Operetta CLS™ High-Content Imaging System (PerkinElmer) or the BZ-9000 (Keyence) microscope.

### High-content image analysis

For image analysis, fixed and stained cells were imaged using a 40x water immersion objective on the Operetta CLS™ High-Content Imaging System (PerkinElmer), allowing automated acquisition of 20–30 image fields per well or insert. Image analysis algorithms were developed for hiPSCs and BCECs, and fluorescence intensity and morphology parameters were quantified using building blocks implemented in the Columbus™ Data Storage and Analysis System (PerkinElmer). Z-stacks were processed for analysis as maximum intensity projections. For the hiPSC analysis algorithm, nuclei were segmented based on Hoechst 33342 staining, border objects were removed, and intensity properties (i.e., mean, min, and max) and basic morphology properties (i.e., area and roundness) were calculated within the selected population of nuclei. Nuclei and fragments with incorrect segmentation were removed by adjusting the previously calculated morphology and intensity properties. The cytoplasm was segmented based on cytoplasm staining or by defining the surrounding region of the already defined nuclei population. The threshold for classifying a cell as positive for a specific marker was set as the mean fluorescence intensity plus three times the standard deviation of the negative control. To segment and quantify the junctional markers in BCECs, filters were applied to reduce noise and continuous background intensity from the images. Subsequently, the Find Image Region building block was used to identify the TJ network (TJP1 and OCLN) or positively stained regions in general (CDH5). Intensity and morphology properties were calculated within these defined regions, and the mean fluorescence intensity was compared in three individual experiments between LOAD and CON cells (mean ± SEM, *n* = 5–6). The results were statistically analysed as indicated in the respective figures.

### Tube forming assay

For the tube forming assay, 24-well plates were coated with 300 µl of undiluted Matrigel for at least one hour at 37 °C. On day 10 of differentiation, BCECs were detached using Accutase for 10 min at 37 °C. After determination of the cell number, 5 × 10^4^ cells/cm^2^ (LOAD BCECs) or 1.5 × 10^4^ cells/cm^2^ (CON BCECs) were seeded in a Matrigel-coated well in endothelial cell medium supplemented with 50 ng/ml vascular endothelial growth factor (VEGF) (Peprotech). The image was captured after 24 h.

### Reconstitution of Aβ-O for transport and cell viability assays

Stabilised soluble Aβ-O (Crossbeta Biosciences) consists of cross-linked Aβ1–42 species, supplied as lyophilised tablets containing 5 µg Aβ-O, 30 mM HEPES, and 200 mM sucrose, were reconstituted in 100 µl H_2_O to obtain a 50 µg/ml Aβ-O solution. To remove excess sucrose, the reconstituted Aβ-O solution was dialysed twice for one hour at room temperature and then overnight at 4 °C against the appropriate cell culture medium using Slide-A-Lyzer™ Dialysis Cassettes (20 K MWCO, 0.5 ml, Thermo Fisher Scientific). A small proportion of the Aβ-O can be presumed to be lost through the dialysis process; nevertheless, this was disregarded when specifying the Aβ-O concentrations used.

For cell viability assays, neurons were treated with Aβ-O concentrations ranging from 30 to 7.5 µg/ml for up to 48 h and hiPSC-derived BCECs were challenged with 7.5 µg/ml Aβ-O for 24 h. For Aβ-O transport assays, BBB integrity was verified by TEER measurements and, on day 10, 7.5 µg/ml Aβ-O in endothelial cell medium was applied to the apical compartment of inserts seeded with BCECs for 24 h.

We have recently published that TEER values exceed 800 Ω*cm^2^ over a 60 h period, together with capacitance values ranging from 1 to 2 µF/cm^2^, indicative of the formation of a tight BCEC monolayer [40]. Here, we chose 48 h post-seeding as the treatment time point for 24 h exposure to 7.5 µg/ml Aβ oligomers, to ensure optimal barrier properties of the model during the transport study.

### Aβ-O transport and quantification

To analyse Aβ-O transport from the apical (top) to the basolateral (bottom) compartment, medium was collected from both compartments. Aβ(1–42) was quantified using the solid-phase enzyme immunoassay Innotest^TM^ β-Amyloid(1–42) (Fijirebio) according to the manufacturer’s instructions. In addition, various Aβ species were identified and subsequently quantified using specific antibodies for monomers, fibrils, and oligomers in a filter retardation assay. For this purpose, samples were spotted directly onto a cellulose acetate membrane using circular templates and filtered through this membrane using a dot blot apparatus. The dialysed Aβ-O solution was used as a positive control, and endothelial cell medium as a negative control. The membrane was washed twice with PBS and dried under vacuum. After blocking the membrane with 1% milk for one hour, the membrane was incubated overnight at 4 °C with the following primary antibodies: Rabbit IgG anti-human Aβ-O (1:1000, #AHB0052, Thermo Fisher Scientific), Mouse IgG anti-human Aβ (1:1000, #803001, Biolegend), or Rabbit IgG anti-human amyloid fibril (1:1000, #ab126468, Abcam). To remove surplus antibodies, the membrane was washed with tris-buffered saline (TBS) and subsequently incubated with the secondary antibody, Goat IgG anti-rabbit HRP (1:20000, #ab6721, Abcam) or Goat IgG anti-mouse HRP (1:10000, #ab6789, Abcam) for two hours at room temperature. The membrane was washed twice for 10 min with TBS-T, followed by a washing step with TBS. Membranes were stained with Amersham™ ECL Select™ (Thermo Fisher Scientific) and were visualised using the ChemiDoc MP Imaging System (Bio-Rad). Aβ-O dots were quantified using Image Lab Software (Bio-Rad), and the ratio of apical to basolateral Aβ was determined in three independent experiments (*n* = 3). Results are shown as mean ± SEM. The results were statistically analysed as indicated in the respective figures.

### Cell viability assay

Cell viability was monitored using the RealTime-Glo™ MT Cell Viability Assay (Promega) according to the manufacturer’s instructions. Briefly, NanoLuc™ luciferase and MT Cell Viability Substrate were dissolved in the appropriate culture medium to obtain a 1x concentration and added to the cells. The luminescent signal produced by metabolically active, and thus live, cells was measured every hour or every two hours using the multiwell plate reader Infinite M1000 Pro (TECAN) in combination with the Fluent 780 robotic station (TECAN) for automation of the process. After four hours, the different treatments were applied to the cells, allowing cell metabolism and assay components to equilibrate and permitting normalisation to each well’s starting signal (four-hour time point). The cytotoxic CellTox™ Lysis Buffer (Promega) served as a positive assay control. BCECs were seeded on white 96-well plates (Greiner bio-one) on day 8 of differentiation and subjected to Aβ-O treatment and cell viability measurement on day 10. Successful differentiation was assumed when BCECs had TEER values > 800 Ω*cm^2^ in parallel experiments. Results are shown as mean ± SEM (*n* = 4); significance was tested with one-way ANOVA followed by Sidak’s multiple comparison test. Neurons were seeded on white 96-well plates (Greiner bio-one) on day 14 after initiation of differentiation from the NPC state. Aβ-O treatment and subsequent measurement of cell viability were then performed on day 30 in functional neurons. The results were statistically analysed as indicated in the respective figures.

### Proteomic analysis

For proteome analysis, hiPSCs and derived BCECs were harvested and lysed using 1x cell lysis buffer (Cell Signaling Technology) containing 1x cOmplete™ Protease Inhibitor Cocktail (Roche). The lysate was incubated for 20 min on ice, and the total protein concentration was measured using the Pierce™ BCA protein assay kit (Thermo Fisher Scientific). Liquid chromatography-mass spectrometry (LC-MS) analysis was performed as described previously [41]. Briefly, samples containing whole cell lysate were alkylated and then separated via sodium dodecyl sulphate polyacrylamide gel electrophoresis. Proteins were stained with Coomassie solution (Serva Electrophoresis GmbH), and protein lanes were cut into small pieces. After de-staining, gel pieces were digested with trypsin (Serva Electrophoresis GmbH) at 37 °C overnight. Extracted peptides were separated with a nano-flow ultra-high-pressure liquid chromatography system (RSLC, ThermoFisher Scientific) equipped with a trapping column and a main separation column (both Acclaim™ PepMap™, Thermo Fisher Scientific). Subsequent MS analysis was performed in data-dependent acquisition mode (DDA) using an LTQ Orbitrap Velos mass spectrometer (Thermo Fisher Scientific), which was coupled to the RSLC system via a Nano Spray Flex Ion Source II (Thermo Fisher Scientific). Samples were first ionised by electrospray ionisation and then fragmented by collision-induced dissociation. Fragment ion mass spectra were recorded in a mass range of 300–1600 m/z. The raw data obtained were processed with Proteome Discovery (Thermo Fisher Scientific) and MaxQuant software, comparing the raw files against reviewed entries of the human UniProt knowledgebase. Proteins were stated as identified by a false discovery rate of 0.01. Overall, 6238 proteins across all groups were identified. In a following filtering step, proteins detected in at least eight samples of one group (hiPSCs or BCECs) were selected, and missing values were imputed. Thus, in total, the label-free quantification (LFQ) intensity of 4902 protein groups was determined.

### Statistical analysis

Statistical analysis was performed using GraphPad Prism Version 7.04 or 9.2.0, and graphics were generated with Adobe Illustrator. Principal component analysis (PCA) was calculated using the Perseus software platform [42]. All experiments were performed at least three times. The level of statistical significance was set as follows: *p < 0.05, **p < 0.01, ***p < 0.001. Detailed information about the individual statistical tests applied can be found in the description of the related methods or in the description of the figures.

## Results

MLUi007-J (LOAD) and MLUi009-A (CON) were used for the in vitro differentiation of BCECs. MLUi007-J was obtained from a 76-year-old female LOAD patient, who carried the AD risk variant APOE4 and had mild grade dementia (Fig. S1A-B). MLUi009-A was obtained from a 64-year-old healthy non-demented control subject, who carried the APOE3 variant unrelated to LOAD and was selected based on similar age, ethnicity, and sex as the patient. HiPSCs exhibited characteristic colony morphology and almost all cells expressed the pluripotency marker proteins OCT4, SOX2, MYC, NANOG, and LIN28A (Fig. S1C-D). DNA methylation analysis of the OCT4 and NANOG promoters revealed no methylation, thus indicating the general activation of the corresponding genes (Fig. S1E).

### BCECs from LOAD HiPSCs resemble an in vitro LOAD BBB model

**Fig. 1:**
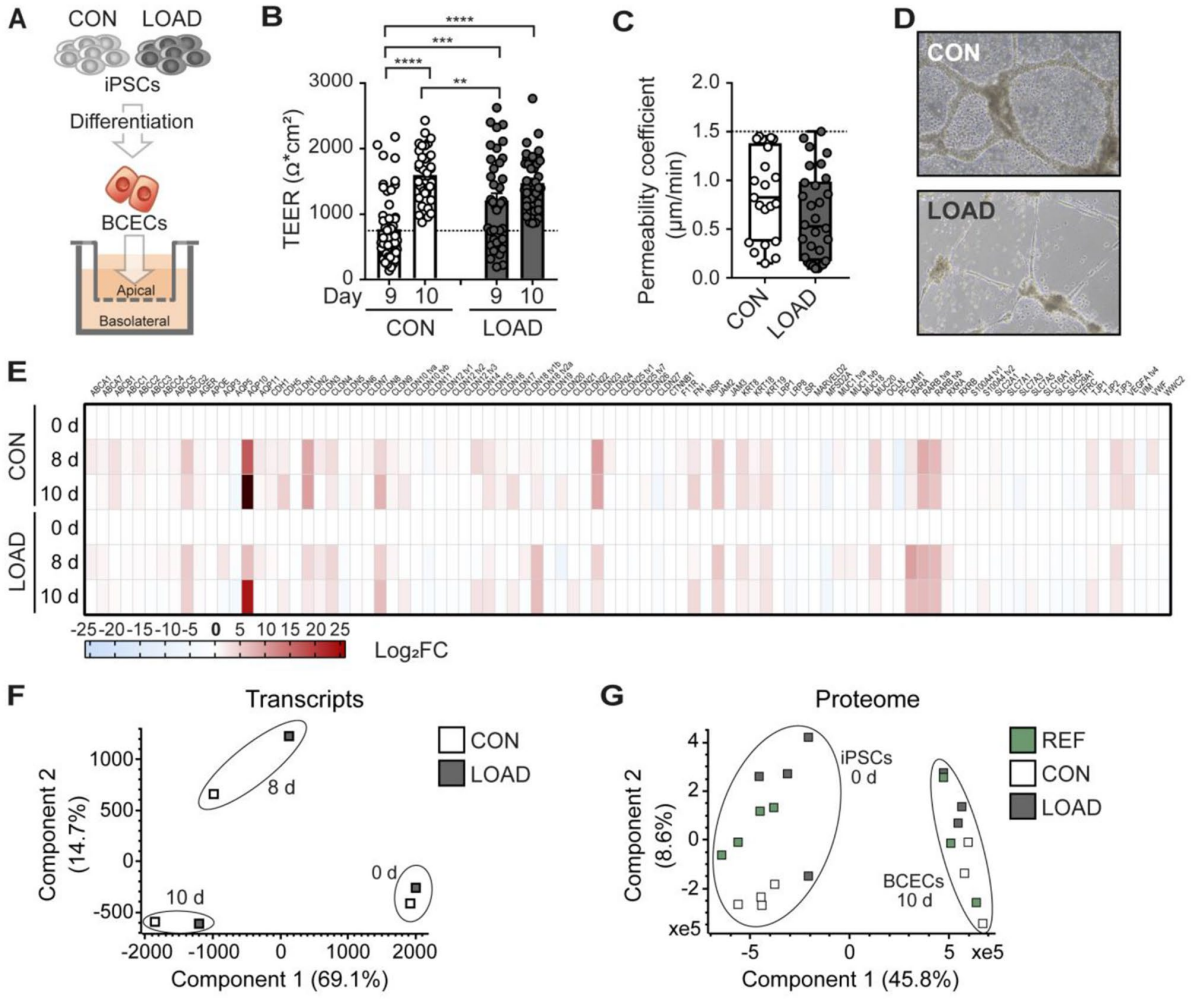
Formation and functional properties of the BBB derived from LOAD-specific hiPSCs. (**A**) Illustration of the experimental setup. hiPSCs derived from a late-onset Alzheimer disease patient (LOAD) and a healthy control individual (CON) were differentiated into brain capillary endothelial-like cells (BCECs) and seeded on insert membranes to form an in vitro blood-brain barrier (BBB). (**B**) Transendothelial electrical resistance (TEER) values measured in the BCEC layer on days 9 and 10 of the differentiation process showed differences in the development of barrier integrity between CON and LOAD models. For all experiments of this study, only inserts with TEER values > 800 Ω*cm^2^ were considered (dotted line). Mean ± SEM of independent experiments (CON *n* = 50, LOAD *n* = 40). Two-way ANOVA following *post-hoc* Tukey’s multiple comparison test, ****p < 0.0001, ***p = 0.0008, **p = 0.0091. (**C**) The permeability coefficient of sodium fluorescein (PC_NaF_) showed a very low permeability and thus hardly any paracellular transport of NaF for CON and LOAD BCECs (PC_NaF_ < 1.5 μm/min). Box and whisker plots displaying independent experiments (CON *n* = 23, LOAD *n* = 30). Unpaired Student’s t-test, *p* = 0.0766. (**D**) The formation of vascular tubes was successful with CON and LOAD BCECs. Scale bar, 100 μm. (**E**) Comparison of CON and LOAD in undifferentiated (0 d) hiPSCs and differentiated BCECs at day 8 (8 d) and day 10 (10 d) by high-throughput multiplex qPCR transcript analysis of BBB markers in independent experiments (CON *n* = 14, LOAD *n* = 12). Log_2_FC for each transcript were plotted on a heatmap as upregulated (red) and downregulated (blue) transcripts compared to 0 d. (**F**) CON and LOAD samples from transcript analyses clustered according to their differentiation stage in a principal component analysis (PCA). (**G**) Proteome analysis of CON and LOAD hiPSCs and BCECs compared to reference (REF) iPSCs and BCECs. A total of 4902 proteins were found in hiPSCs (*n* = 4) and BCECs (*n* = 3) in independent experiments. PCA found that hiPSCs and BCECs are clustered according to their differentiation stage

**Fig. 2:**
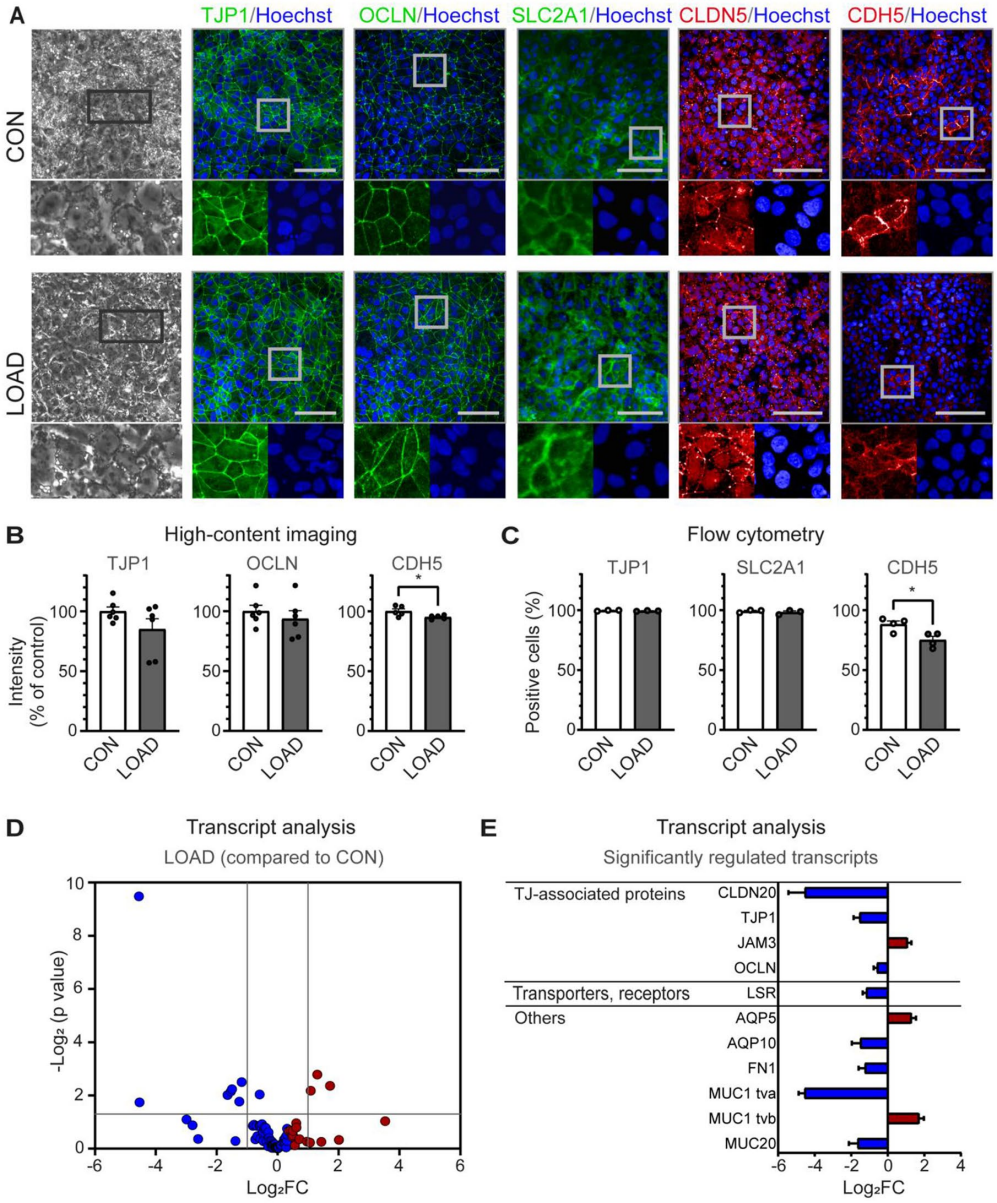
LOAD-specific regulation of transcripts and proteins in BCECs. (**A**) Phase-contrast and immunofluorescence images of CON and LOAD BCECs confirmed the development of a continuous cell monolayer that exhibited the characteristic expression and localisation of relevant BBB protein markers at day 10 of differentiation. Scale bar, 100 μm. (**B**) High-content image analysis in BCECs revealed that CDH5 levels were decreased in LOAD compared with CON. Mean ± SEM of *n* = 5–6 independent experiments. Unpaired Student’s t-test, *p = 0.0384. (**C**) Quantification of TJP1, SLC2A1, and CDH5 in BCECs by flow cytometry confirmed a significant reduction in CDH5 in LOAD. Mean ± SEM of *n* = 3–4 independent experiments. Unpaired Student’s t-test, *p = 0.0183. (**D**) Comparison of CON and LOAD BCECs by high-throughput multiplex qPCR transcript analysis of BBB markers in independent experiments. Log_2_FC values for each transcript were plotted against *p*-values (Student’s t-test, *p* ≤ 0.05) in a volcano plot with downregulated (blue) and upregulated (red) transcripts displaying the independent experiments performed (CON *n* = 14, LOAD *n* = 12). (**E**) List of significantly regulated BBB markers (mean + SEM)

To study the link between AD and BBB dysfunction, LOAD and CON hiPSCs were used for the in vitro differentiation of BCECs. The hiPSC line WISCi004-B served as an additional reference line (REF), as previously described by our group for the successful production of BCECs [25] (Fig. S2A-C). For the in vitro differentiation of BCECs, hiPSCs were first differentiated in culture dishes and then seeded as progenitor cells on insert membranes to create an in vitro BBB model based on a static Insert setup (Fig. 1A). LOAD, CON, and REF hiPSC-derived BCECs developed an increasingly strong barrier after nine days of differentiation, which was strongest at day 10, with TEER values of approximately 1500 Ω*cm^2^ (Fig. 1B, Fig. S2D). TEER values were higher (*p* = 0.0008) on day 9 of differentiation in LOAD BCECs compared with CON BCECs, suggesting LOAD-specific differences during the formation of the barrier by cell-cell contacts. The strong barrier of LOAD, CON, and REF BCECs was also verified by a limited paracellular permeability of NaF (Fig. 1C, Fig. S2E). Both LOAD and CON BCECs were able to build vascular tube-like structures in the presence of VEGF as a characteristic feature of endothelial cells (Fig. 1D). Then, transcriptional changes in the BBB-associated transcripts were monitored by high-throughput multiplex qPCR analyses during the course of differentiation in LOAD, CON and REF BBB differentiation models (Fig. 1E, Fig. S2F). During the differentiation of hiPSCs into BCECs, a broad spectrum of transcripts was induced, including several claudins, transcripts of junctional and structural proteins of the paracellular space, as well as relevant transporters and receptors. LOAD and CON cells showed similar transcriptional profiles over the differentiation process, as both LOAD and CON samples clustered according to their differentiation stage (hiPSCs at 0 d, progenitors at 8 d, BCECs at 10 d) in PCA (Fig. 1F). The next question was how comparable the transcription profile of REF BCECs is to that of LOAD and CON BCECs. REF BCECs showed a largely similar expression profile with few differences (logarithm of fold change; log_2_FC > 2) depending on the differentiation stages (Fig. S2G). This observation was confirmed in a proteomic analysis that included LOAD, CON and REF BBB differentiation models. The analysis of 4902 proteins also revealed that LOAD, CON, and REF clustered according to their differentiation stage (hiPSCs at 0 d, BCECs at 10 d) in PCA (Fig. 1G). Both LOAD and CON BCECs exhibited the typical cobblestone-like cell morphology (Fig. 2A). Additionally, we monitored the expression and localisation of endothelial-specific marker proteins by immunofluorescence staining. TJP1, OCLN, and CLDN5 indicated the formation of a continuous TJ network in the differentiated BCECs. TJP1, OCLN, and SLC2A1 were predominantly found at the cell boarders, whereas CLDN5 and CDH5 were mostly enriched intracellularly (Fig. 2A). High-content imaging showed no notable differences in TJP1 and OCLN, but significantly reduced levels of CDH5 in LOAD BCECs compared with CON cells (*p = 0.0384) (Fig. 2B). Quantification of TJP1, SLC2A1, and CDH5 by flow cytometry analysis confirmed the significant decrease in CDH5 (*p = 0.0183) (Fig. 2C). We extended our analysis of BBB-specific endothelial cell markers for the comparison of LOAD and CON BCECs using the same high-throughput multiplex qPCR as previously mentioned. This analysis revealed eight significantly downregulated and three significantly upregulated BBB-related transcripts. The dataset contained reduced expression of TJ-associated transcripts, including CLDN20, TJP1, and OCLN, as well as the lipolysis-stimulated lipoprotein receptor (LSR) and other transcripts, such as aquaporin 10 (AQP10), fibronectin (FN1), and mucins (MUC1 tva, MUC20), in LOAD BCECs. In contrast, functional adhesion molecule C (JAM3), aquaporin 5 (AQP5), and MUC1 tvb were significantly increased in LOAD BCECs. Interestingly, MUC1 showed both significant up- and downregulation depending on the transcript variant studied (Fig. 2D-E). We then compared these results with REF BCECs, which expressed transcripts that diverged from both LOAD and CON BCECs (Fig. S2G). These included transcripts that were found to be LOAD-associated compared with CON (CLDN 20 and AQP5) and transcripts that were not LOAD-regulated compared with CON (CLDN23, JAM2, and RARA).

### Interlaboratory comparison of the in vitro LOAD BBB model

**Fig. 3:**
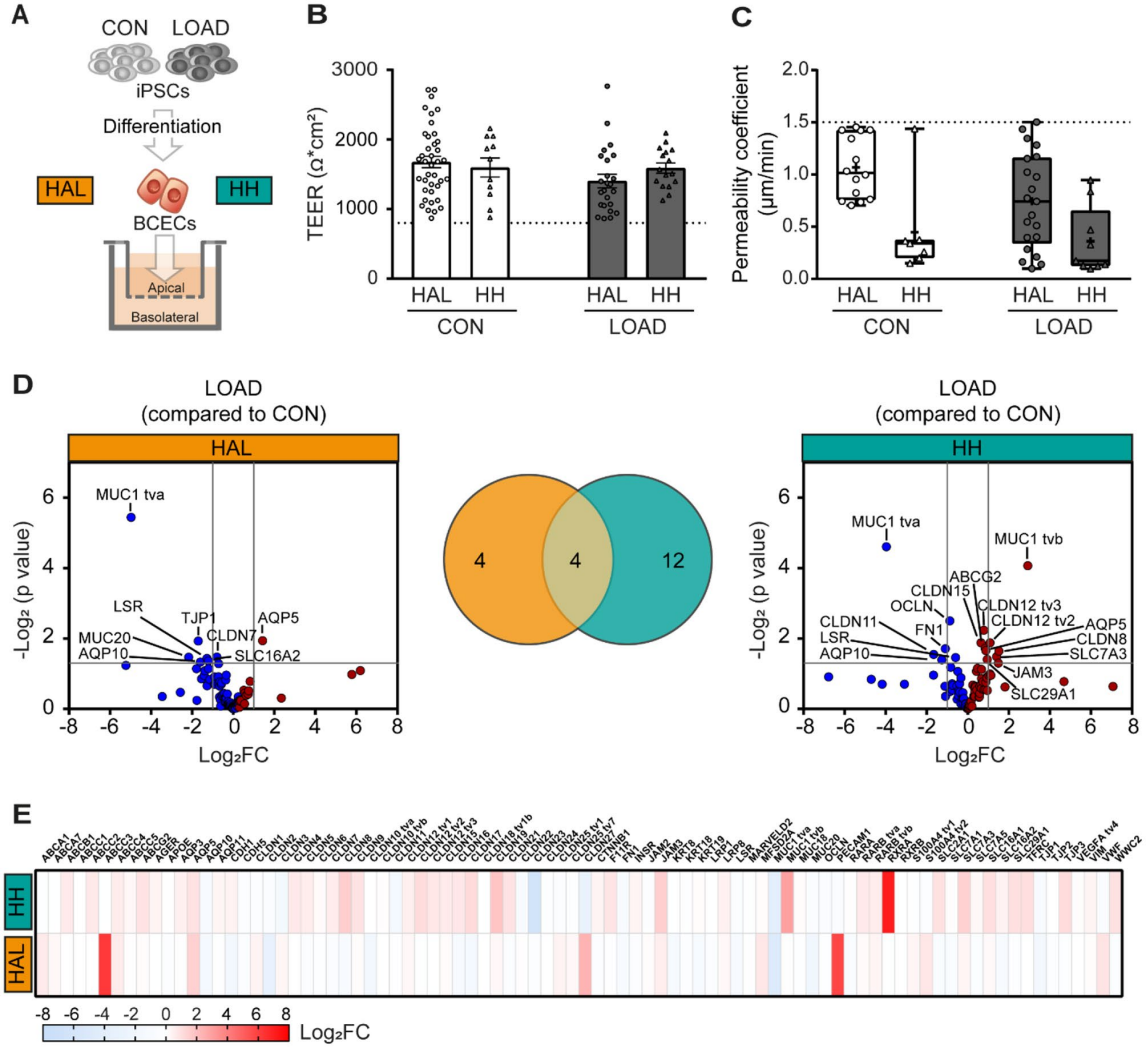
Analysis of the transferability of the in vitro BBB model between two independent laboratories. (**A**) The experiments were performed according to standardised protocols in two independent laboratories in Halle (HAL) and Hamburg (HH) and compared to confirm the transferability of the in vitro BBB model. (**B**) Transendothelial electrical resistance (TEER) values measured in the BCEC layer on day 10 of the differentiation process demonstrated an intact barrier for CON and LOAD BCECs in both laboratories. For all experiments of this study, only inserts with TEER values > 800 Ω*cm^2^ were considered (dotted line). Mean ± SEM of independent experiments (CON: *n* = 39 HAL, *n* = 11 HH; LOAD: *n* = 24 HAL, *n* = 16 HH). Two-way ANOVA following *post-hoc* Tukey’s multiple comparison test revealed no significant diffferences. (**C**) The permeability coefficient of sodium fluorescein (PC_NaF_) showed a very low permeability and thus hardly any paracellular transport of NaF for CON and LOAD BCECs (PC_NaF_ < 1.5 μm/min) in both laboratories. Box and whisker plots displaying independent experiments (CON: *n* = 14 HAL, *n* = 7 HH; LOAD: *n* = 21 HAL, *n* = 9 HH). Unpaired Student’s t-test, *p* = 0.0766. (**D**) Both laboratories jointly performed high-throughput multiplex qPCR transcript analysis to compare key BBB markers (CON: *n* = 9 HAL, *n* = 5 HH; LOAD: *n* = 7 HAL, *n* = 5 HH). Log_2_FC values for each transcript were plotted against *p*-values (Student’s t-test, *p* ≤ 0.05) in a volcano plot with downregulated (blue) and upregulated (red) transcripts for both laboratories. Four transcripts were significantly altered in LOAD in both laboratories, as shown in a Venn diagram. (**E**) Log_2_FC values for each transcript in LOAD BCECs compared to CON BCECs were plotted on a heat map for comparison of Log_2_FC values of LOAD BCECs between both laboratories (*n* = 5–9 for HAL or HH in independent experiments)

To evaluate the consistency and reliability of results, we compared the barrier integrity and the transcriptional profiles of in vitro LOAD and CON BBB models obtained from different laboratories. Despite minor procedural differences, TEER measurements confirmed the formation of an intact barrier on day 10 of differentiation in both models generated across four independent laboratories (Fig. S3). A detailed analysis of an extended dataset from two laboratories (Halle [HAL] and Hamburg [HH]) further revealed no significant differences in TEER values (CON *p* = 0.6458, LOAD *p* = 0.1668) (Fig. 3B). Furthermore, paracellular permeability measurements using NaF confirmed the integrity of the barrier on day 10 of differentiation for LOAD and CON BCECs from HAL and HH (Fig. 3C).

Next, this study compared the transcriptional profile and found differences between the two laboratories. High-throughput multiplex qPCR analysis of the BBB-associated markers revealed 16 (HH) and 8 (HAL) significantly differently regulated transcripts, respectively, for the LOAD BBB model compared with CON BCECs (Fig. 3D-E). LOAD BCECs from HH showed a significant downregulation of six transcripts, including AQP10, CLDN11, FN1, OCLN, LSR, and MUC tva. Furthermore, upregulation of 10 transcripts was detected, including several claudins (CLDN8, -12 tv2, -12 tv3, and − 15), TJ-associated JAM3, various transporters (ABCG2, SLC7A3, and SLC29A1), AQP5, and MUC1 tvb. In contrast, LOAD BCECs from HAL showed a low number of transcripts that were significantly regulated. There was a significant increase in AQP5 and a significant decrease in seven transcripts, including AQP10, LSR, CLDN7, TJP1, SLC16A2, and mucins (MUC1 tva, MUC20). Four significant changes were observed in both laboratories, including an increase in AQP5 and a decrease in the expression levels of LSR, AQP10 and MUC1 tva.

In summary, the transcriptional profile of LOAD BCECs for the BBB-associated markers is largely, but not entirely, consistent between the two laboratories. Four significant changes were confirmed in the interlaboratory comparison of the in vitro LOAD BBB model.

### Challenging the in vitro BBB model with Aβ-O

**Fig. 4:**
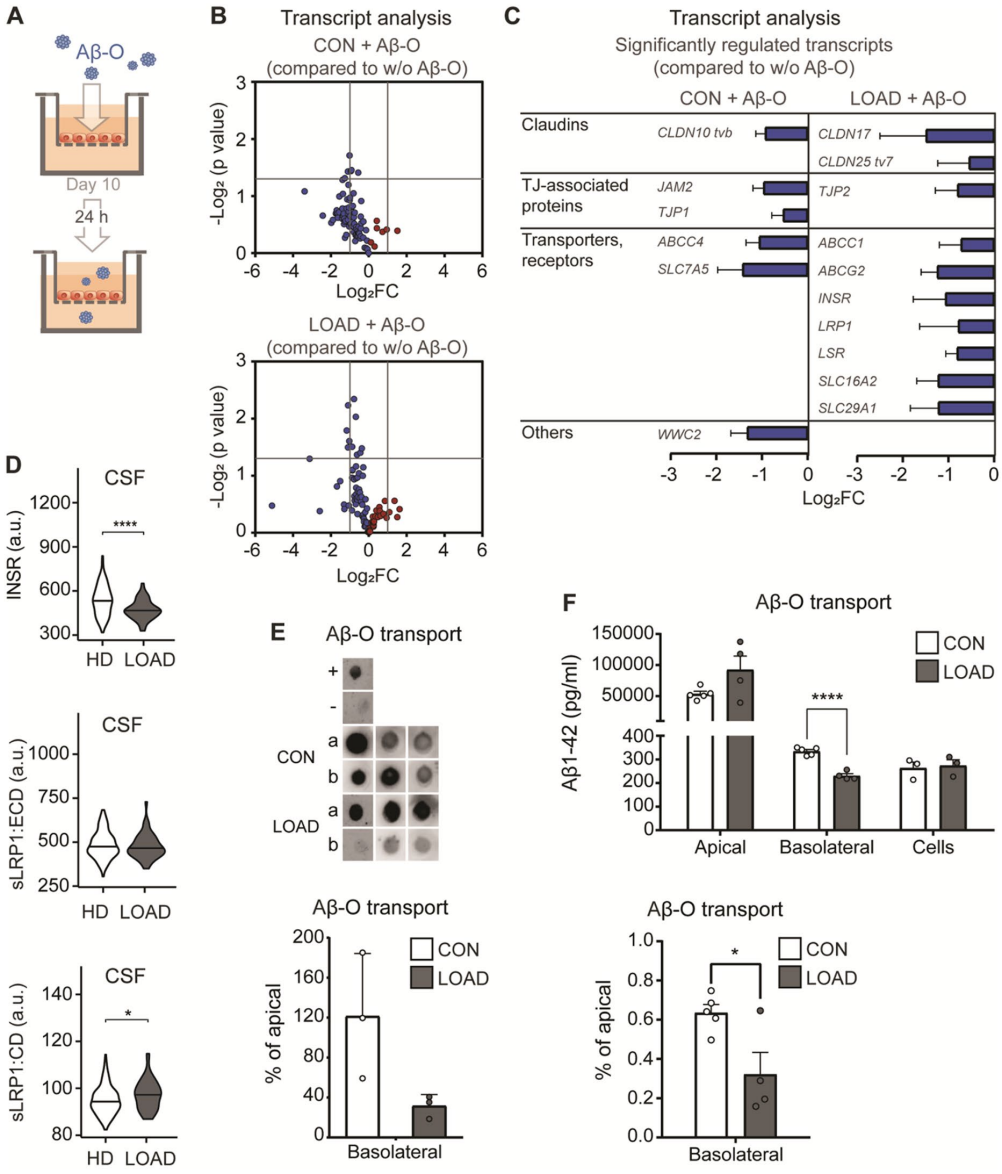
Amyloid-β oligomer transport and induced transcriptional changes in the in vitro BBB. (**A**) Visualisation of the experimental setup of the in vitro LOAD BBB model. We only included BCECs with TEER values > 800 Ω*cm^2^ on day 10 for apical treatment with amyloid-β oligomers (Aβ-O; 7.5 µg/ml) for 24 h. (**B**) Comparison of Aβ-O-treated CON and LOAD BCECs by high-throughput multiplex qPCR transcript analysis of BBB markers in independent experiments. Log_2_FC values for each transcript were plotted against *p*-values (Student’s t-test, *p* ≤ 0.05) in a volcano plot with downregulated (blue) and upregulated (red) transcripts displaying *n* = 3–4 independent experiments performed. (**C**) List of significantly regulated BBB markers (mean + SEM). (**D**) Relative protein levels of INSR, LRP1 (extracellular domain, ECD; cytoplasmic domain, CD) in CSF from a human cohort comprising 148 healthy donors (HD, APOE3 carriers) and 51 individuals with LOAD and confirmed amyloid pathology (APOE4 carriers). Results are presented as relative levels (Mean, Student’s t-test, ****p < 0.0001, *p = 0.48). (**E**) Analysis of Aβ transport in response to Aβ-O treatment by the filter retardation assay. The raw data for the dot blot are shown at the top, and the quantification is shown below. Plot of the ratio of apical (a) and basolateral (b) Aβ in the respective in vitro CON and LOAD BBB models (mean ± SEM, *n* = 3). (**F**) Quantification of Aβ transport in response to Aβ-O treatment using an Aβ(1–42)-specific ELISA performed for *n* = 4–5 independent experiments (Mean ± SEM; Unpaired Student’s t-test, ****p < 0.0001, *p = 0.024)

Next, the impact of the observed changes in vascular transcripts and transport pathways on the functionality of the in vitro BBB models was investigated by challenging them with cytotoxic Aβ-O. We wanted to know whether the transport of Aβ-O from the apical compartment (mimicking the vascular lumen) across the barrier of BCECs to the basolateral compartment (mimicking the CNS) is altered in LOAD (Fig. 4A).

First, a physiologically relevant concentration of our synthetic Aβ-O was determined for our experiments. For this purpose, hiPSC-derived neurons were used as a sensitive cell type in the CNS, which is predominantly degenerated in Alzheimer disease. The cell viability of neurons was significantly reduced (****p < 0.0001) in a time- and dose-dependent manner upon treatment with 7.5, 15, and 30 µg/ml Aβ-O for 24 h and 48 h (Fig. S4A-B). According to this observation, we examined treatment with 7.5 µg/ml Aβ-O for 24 h in LOAD and CON BCECs, finding it did not affect cell viability (Fig. S4C-D). Treatment with 7.5 µg/ml Aβ-O for 24 h did not affect barrier integrity as measured by TEER and NaF permeability in LOAD and CON BCECs (Fig. S4E-F). Notably, the applied Aβ-O solution contained not only soluble oligomers but also other Aβ species, such as Aβ peptides, monomers, and fibrils (Fig. S4G).

We investigated the impact of 7.5 µg/ml Aβ-O treatment on the panel of BBB-associated transcripts; it did not increase any transcripts but decreased many in LOAD and CON BCECs. CON BCECs revealed significantly decreased levels of the TJ-associated transcripts CLDN10 tvb, TJP1, and JAM2, as well as reduced levels of ABCC4, SLC7A5, and WWC2. LOAD BCECs showed a significant decrease in the TJ transcripts CLDN17, CLDN25 (tv7), and TJP2, as well as reduced levels of various transporters and receptors, including ABCC1, ABCG2, INSR, LRP1, SLC16A2, and SLC29A1, in response to Aβ-O treatment (Fig. 4B-C).

To validate the in vitro data set obtained, data on protein expression in the CSF from the ADNI cohort were analyzed. Protein data from 148 APOE3/3 carriers without any Aβ or tau pathology were compared to 51 APOE4 carriers with LOAD and Aβ deposits in the brain, as detected by PET imaging. Notably, this in vivo human data revealed a significant reduction in INSR in individuals with LOAD, while the expression of the extracellular domain of LRP1 (sLRP1:ECD) was increased and the cytoplasmic domain of LRP1 (sLRP1:CD) remained unchanged (Fig. 4D).

The addition of Aβ-O to the apical compartment of the BBB insert model also allowed the assessment of Aβ-O transport across the BCEC barrier. This analysis revealed reduced transport of Aβ-O across the barrier of LOAD BCECs in comparison with CON BCECs, as indicated by the filter retardation assay (Fig. 4E). A significantly (*p = 0.024) reduced transport of Aβ was confirmed by a quantitative Aβ(1–42)-specific ELISA. (Fig. 4F). These analyses using two different techniques demonstrated that Aβ transport was reduced in the in vitro LOAD BBB model.

Together, these results demonstrate that BCECs from LOAD hiPSCs resemble an in vitro LOAD BBB model with an intact but altered endothelial barrier and a LOAD-specific expression profile of BBB-associated transcripts. Challenging the in vitro BBB model with Aβ-O affected the expression of BBB-associated transcripts in a LOAD-specific manner, reducing Aβ-O transport only in LOAD without affecting barrier tightness.

## Discussion

This study presents an in vitro human LOAD-specific BBB model using fully characterised hiPSCs obtained from LOAD dementia patients [27]. The barrier properties of BCECs derived from LOAD hiPSCs carrying the AD risk variant APOE4/4 were thoroughly examined and compared to those from CON hiPSCs with the APOE3/3 variant, along with the established REF line [43]. All BCECs exhibited typical vascular and CNS barrier characteristics as they formed tube-like structures and established a tightly sealed monolayer with limited paracellular transport of NaF and high TEER values around 1500 Ω*cm^2^. Importantly, these TEER values are consistent with those reported in previous hiPSC-derived BBB models [27, 44, 45] and are comparable to in vivo measurements, ranging between 500 and 6000 Ω*cm^2^. Notably, we observed that TEER values were significantly higher on day 9 of differentiation in LOAD BCECs compared with controls, indicating LOAD-specific differences in the development of endothelial cells and the formation of cell-cell contacts.

This study conducted a comprehensive analysis of the transcriptional profile of healthy and diseased hiPSC-derived BCECs, focusing on a targeted selection of key BBB transcripts essential for barrier maintenance (CLDN5, TJP1, and OCLN) and transport processes (SLC2A1, ABCB1, and LSR) [28]. Given their pivotal role in maintaining BBB homeostasis, these transcripts are vital for evaluating BBB integrity, particularly in relation to changes associated with LOAD. While human brain tissue transcriptome or single-cell sequencing studies may offer larger datasets, they often lack the sensitivity to detect the BBB markers that we have examined [46, 47]. These datasets may confirm or contradict some of our findings; however, it is important to note that the diverse endothelial cell types in the brain are difficult to distinguish using current dissection techniques or marker expression patterns, leading to uncertainty about the specific cell types being analysed. In contrast, our hiPSC-derived endothelial cells represent a homogenous population of BCECs that mimic the microvascular endothelial cells of brain capillaries.

Following the course of differentiation from hiPSCs to BCECs, we aimed to analyse cellular transition and barrier establishment across different hiPSC lines. This study combined transcript analysis with a proteomic analysis of 4902 proteins, which revealed little variation among hiPSCs, progenitors, and differentiated BCECs across the LOAD, CON, and REF groups. The grouping of cell types according to their differentiation status underscores the robustness of the differentiation process and confirms that the disease background does not impede in vitro development into BCECs, as seen in other hiPSC models studying neurodegenerative diseases [48]. Nonetheless, comparison of the widely used REF line to LOAD and CON BCECs revealed differences regarding a small number of transcripts. These differences may arise because LOAD and CON BCECs were derived from hiPSCs of older individuals (> 60 years), whereas REF BCECs originated from prenatal somatic tissues [27, 49]. Despite matching for ethnicity and sex across all hiPSCs, ongoing investigations aim to determine which characteristics of the donor’s age persist in hiPSCs. Molecular ageing causes changes in DNA, including genomic instability, telomere shortening, and mutations [50]. This raises the question of how these changes influence hiPSCs derived from older donors [51], emphasising the importance of using age-matched control cells, as implemented in our study.

Analysis and direct comparison of BCECs derived from LOAD and CON hiPSCs revealed the characteristic expression and localisation of BBB-associated junction and transporter proteins (TJP1, OCLN, SLC2A1, and CLDN5). In line with this, we observed reduced LSR transcript expression and CDH5 protein expression, both of which are relevant to maintaining BBB integrity and function. LSR plays a multifaceted role, not only as a component of tricellular junctions [52] but also in maintaining brain cholesterol homeostasis [53] and related Aβ mechanisms [54], both critical in AD (reviewed in [55]). CDH5 is an endothelial cell-specific cadherin and the main component of adherence junctions (AJ), which are involved in cell-cell adhesion and barrier integrity [56]. While most studies have focused on TJs, few have investigated AJs in the context of brain disease; therefore, their role in AD-related cerebrovascular pathology is not well understood. Consistent with our data, a study in APP/PS1 mice - a mouse model for studying amyloid pathology - indicated a disruption in CDH5 expression that occurred before plaque formation and BBB breakdown [57]. Only recently, CDH5 has been identified as a marker of endothelial injury in preclinical AD when analysing the CSF of AD brains [58]. Similarly, elevated plasma levels of CDH5 and reduced levels in brain capillaries have been found in a mouse model and patients with AD or MCI [59]. The study further proposes that Aβ (1–42) peptides induce the cleavage and release of the ectodomain of CDH5, resulting in increased levels of soluble CDH5 and vascular impairment [59]. Elevated levels of soluble CDH5 have also been implicated in BBB breakdown in the context of inflammation [60, 61]. Moreover, the internalisation of this highly dynamic molecule has been proposed to reduce CDH5 at cellular junctions, compromising barrier function [56]. Our results further demonstrate that the genetic LOAD background is sufficient to induce a CDH5 phenotype in BCECs and that the AD-related reduction in CDH5 can be modelled in vitro in a hiPSC-based version of the human BBB.

Additionally, our analysis of aquaporins revealed significant regulation in LOAD BCECs, with AQP5 upregulated and AQP10 downregulated. Aquaporins play a crucial role in facilitating selective water transport across cell membranes, which is vital for maintaining osmotic balance in cells and tissues [62]. While previous research primarily focused on AQP4 in the context of AD [63, 64], our findings indicate that AQP5 may also be relevant to disease pathology, particularly given its upregulation in the cerebral cortex of APP/PS1 mice [65]. AQP10 belongs to the aquaglyceroporin subfamily, allowing the transport of water, glycerol, and small solutes. To our knowledge, it has not been previously linked to AD, but its role in lipid metabolism may be relevant to the disease [64, 66].

Mucins, a protein family rarely investigated in AD, were differentially expressed in LOAD BCECs, with MUC1 (tvb increased, tva decreased) and MUC20 (decreased) showing significant regulation. These O-glycosylated, membrane-bound proteins are typically present on epithelial and endothelial cells [67]. We previously reported that altered glycation in transfected human brain microvascular endothelial cells (THBMECs) contributes to barrier dysfunction [68]. In a recent publication, we highlighted the relevance of mucin regulation in a LOAD co-culture model of the NVU [26], discussing its potential as an ageing marker and its involvement in angiogenesis, inflammation, and AD [26]. Mucins have recently drawn attention in AD research due to their dysregulation during ageing and in neurodegenerative diseases [69]. The authors demonstrated that ageing- and disease-related changes in brain endothelial mucin-domain glycoproteins disrupt BBB function, potentially leading to brain haemorrhaging in mice. They also demonstrated that they could improve BBB function and reduce neuroinflammation and cognitive deficits in aged mice by restoring core 1 mucin-type O-glycans to the brain endothelium, indicating a potential druggable target related to our findings.

We observed no significant changes in ABCB1 or ABCG2. However, these transporters are often altered in Alzheimer disease and related models. ABCB1 may not have been directly influenced by our APOE4/4 haplotype, while changes in ABCG2 have been described differently [70].

To evaluate the robustness of the proposed LOAD BBB model, an interlaboratory comparison of results obtained from the in vitro LOAD BBB model was conducted across four independent laboratories. The formation and integrity of the barrier were assessed using the same standardized protocol, albeit with minor variations. Despite these differences, a consistently robust generation of in vitro BBB models with comparable tightness was observed across all laboratories. Further analysis of transcriptional changes from two different laboratories confirmed increased expression of AQP5, as well as reduced levels of LSR, AQP10, and MUC1 tva, although some other changes were not consistently validated. Numerous factors can affect the reliability of studies and the reproducibility of results (reviewed in [71]). The confirmed regulation of AQP5, AQP10, LSR, and MUC1 tva indicates a strong effect on the transcriptional regulation of these genes. Transcripts identified as significant in the joint analysis of all samples may have lost significance due to interlaboratory variability and the low level of regulation. Mitigating interlaboratory variability to achieve high comparability between studies and across different laboratories remains a critical challenge for the in vitro LOAD BBB model.

This was accompanied by the identification of multiple differentially regulated BBB-associated transcripts, indicating disturbed barrier function. Intriguingly, the Aβ-O applied proved toxic to hiPSC-derived neurons, yet it had no discernible impact on cell viability, TEER, or NaF permeability of BBB-forming BCECs. While few studies reported drastic effects at the BBB, such as cell loss or altered tightness, in response to Aβ treatment [72, 73], others primarily observed impaired Aβ clearance through differentially expressed transporters or Aβ accumulation at the BBB [47, 74–77]. Aberrant production and impaired clearance of Aβ peptides, leading to subsequent deposition in the brain, constitute a central and early factor in the pathology of AD. In particular, the APOE4/4 background of our sporadic LOAD BCECs is associated with increased vascular Aβ depositions and plaque formation in LOAD patients [78, 79]. In vivo microdialysis experiments in hAPP-transgenic mice expressing human APOE isoforms demonstrated an APOE4-dependent decrease in brain clearance of soluble Aβ, reflected in elevated Aβ depositions in these mice [80]. Our current understanding is derived mainly from animal models with genetic modifications linked to familial AD. While a few reports on hiPSC-derived BBB models also highlight altered Aβ metabolism and transport [72, 81], they only focus on cells derived from early-onset familial AD cases. Intriguingly, our sporadic LOAD models reflected these findings to some extent, as the amount of Aβ reaching the basolateral compartment after apical administration was reduced in the diseased BBB. The Aβ level within the BCEC layer, however, remained unaffected by the genetic background. Recent findings support this, demonstrating that the APOE4-related increase in Aβ accumulation in hiPSC-derived BBB models depends on the presence of mural cells [75]. Notably, soluble Aβ-O have demonstrated their impact in various disease models by reducing synaptic density, enhancing long-term synaptic depression, and inducing hyperphosphorylation of tau [82]. The toxicity of Aβ is known to be cell type-specific [83] and is also influenced by the diverse amyloid species and aggregation forms present [84]. The Aβ-O solution we used contained not only soluble oligomers but also Aβ monomers and fibrils, and therefore reflects the effects of different Aβ species.

When treating the apical side of our LOAD-BBB model with Aβ-O, we observed less basolateral Aβ-O compared to the control BBB model. Apical administration of Aβ-O mimics the transport of Aβ-O from the blood side into the CNS. Our observation of less transport of Aβ-O can be explained by the downregulation of several Aβ-O transporters such as LRP1, LSR, ABCC1, and ABCG2, whereas AGER remained unchanged. The altered transporters respond immediately to Aβ-O in order to eliminate this substance from the CNS, with only small amounts of Aβ-O being transported back into the CNS [85–88]. AGER is induced with a delay in LOAD and increasingly transports Aβ-O into the CNS [89]. We do not see increased transport of Aβ-O, we observed less Aβ-O transport. The results therefore show the immediate effect on LRP1, LSR, ABCC1, and ABCG2 and support the hypothesis that AGER induction is delayed until Aβ-O deposits, inflammation, and permeability disorders have progressed. This in vitro model is therefore novel in that it models not clearance from the CNS, but rather the equally pathological transport back into the CNS.

The exact mechanisms underlying the altered transport of Aβ across the BBB remain elusive in our measurements, necessitating additional research for a comprehensive understanding. Nevertheless, Aβ-O treatment induced various alterations at the transcriptional level, with specific implications in Aβ regulation. Notably, the downregulation of key receptors and transporters, such as LRP1, LSR, and INSR, following Aβ-O exposure was observed exclusively in LOAD models. In contrast, our CSF protein data from LOAD patients showed an increase in the extracellular domain of LRP1, while the cytoplasmic domain remained unchanged. Since LRP1 acts as a lipid receptor with two domains and is expressed in various brain cell types, its expression levels may be differentially affected by AD depending on the specific cell type or domain involved [90]. Despite conflicting reports and our contrary results, the pivotal role of the lipid receptor LRP1 in AD, as well as APOE4 mechanisms, is evident and largely associated with compromised Aβ clearance from the brain to the bloodstream [47, 74, 90]. Cerebrovascular AD pathology includes a metabolic component characterised by impaired glucose uptake and insulin response. Alterations of INSR in AD are associated with changes in glucose transport [91], although we did not observe any immediate effects on SLC2A1. The insulin receptor INSR is diminished in the brain vasculature of individuals diagnosed with AD and correlates with Aβ accumulation in the brain [92]. We showed a reduction in INSR in the CSF of a human LOAD cohort with an APOE4 background [93], emphasising the findings in our hiPSC model. Furthermore, Aβ promotes the degradation of INSR in brain capillaries [94] and competes with insulin for binding to INSR to block INSR signalling [95], collectively contributing to insulin resistance in AD.

In summary, our results emphasise the multifaceted effect of Aβ on the LOAD BBB. LOAD and CON BCECs not only differ in the expression of BBB-associated transcripts and proteins, but they also respond differently to Aβ-O administration. We demonstrated that LOAD BCECs regulate other transcripts in response to Aβ-O administration and are less able to shuttle Aβ-O. Thus, we provide a human model for future basic and applied research to develop therapeutic strategies for LOAD.

## Supplementary Information

Below is the link to the electronic supplementary material.

Supplementary Material 1

## Data Availability

The authors confirm that the data supporting the findings of this study are available within the article and its supplementary materials. Raw data supporting the findings of this study are available from the corresponding author upon request.
