# Supplementary material for: Brain capillary endothelial-like cells show altered barrier functionality and reduced transport of amyloid β in late-onset Alzheimer disease
Source: Fluids Barriers CNS. 2026 Jan 22;23:11. doi: 10.1186/s12987-025-00753-7 (PMC12829042; doi:10.1186/s12987-025-00753-7)
Supplement: Supplementary file 1 — Supplementary Material 1 [file 12987_2025_753_MOESM1_ESM.docx]

**Additional file**

**Title**

**Brain capillary endothelial-like cells show altered barrier functionality and reduced transport of amyloid β in late-onset Alzheimer disease**

Carla Hartmann^#,1^, Undine Haferkamp^#,2^, Antje Appelt-Menzel^3,4^, Janica Barenberg^2^, Andreas Brachner^5^, Toni Ehrhard^1^, Julia Feldhaus^1^, Anna Gerhartl^5^, Thomas Hollemann^1^, Linda Anna Michelle Kulka^1^, Selin Leckzik^1^, Jennifer Leu^2^, Marcel Seungsu Woo^11,12^, Manuel Alexander Friese^12^, Alzheimer’s Disease Neuroimaging Initiative*, Marco Metzger^3,4^, Winfried Neuhaus^5,6^, Sabrina Oerter^3,4^, Heidi Olzscha^1,7^, Andreas Pich^8^, Dagmar Riemann^9^, Ole Pless^2^, Dan Rujescu^10^, Matthias Jung^1,13^

**Inventory of additional file 1**

Figure S1

Figure S2

Figure S3

Figure S4

Table S1

Table S2

Methods additional file

References additional file

# **Figure S1**


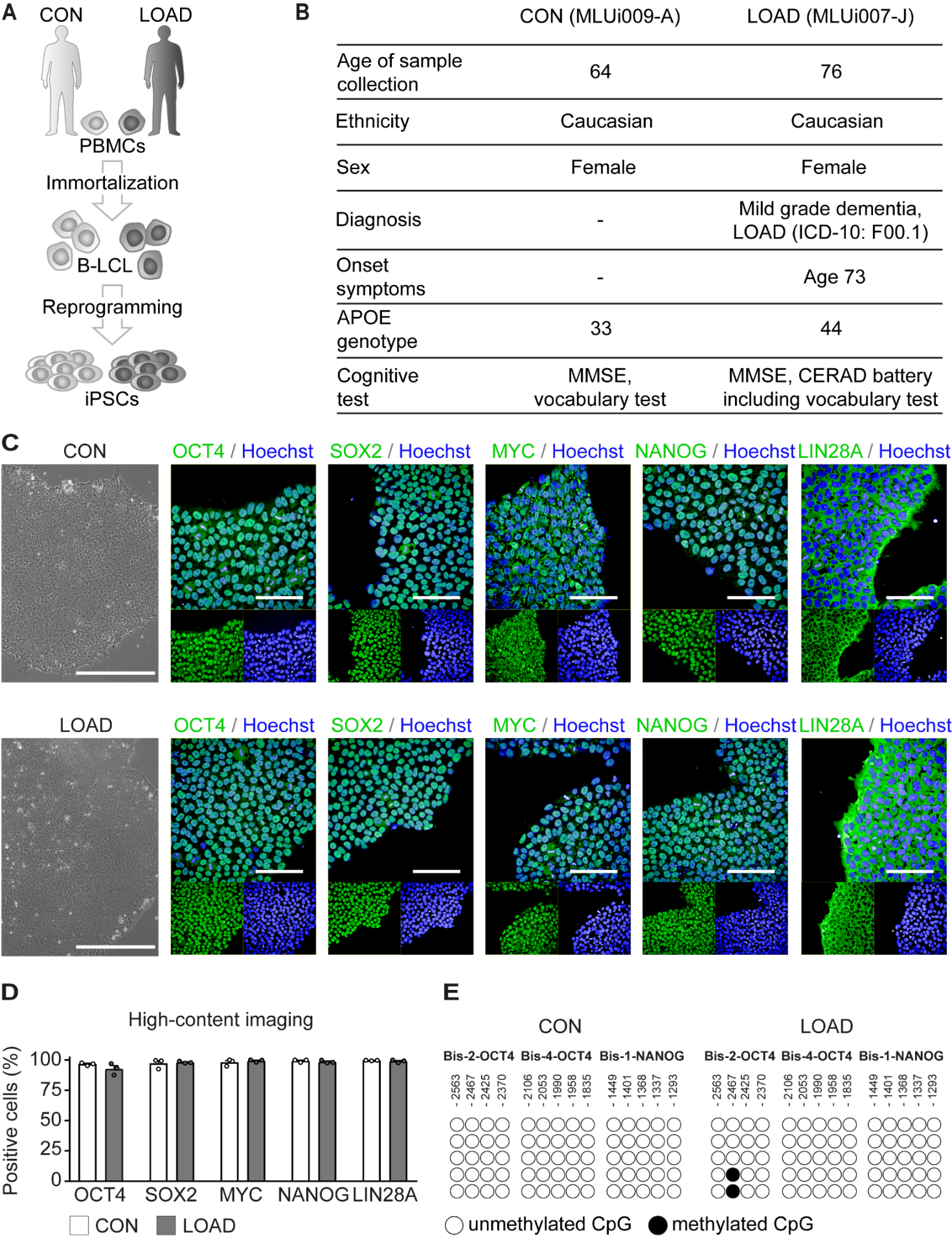


**Generation and characterization of the hiPSC lines MLUi009-A and MLUI007-J.** (**A**) Experimental setup for the generation of human induced pluripotent stem cells (hiPSCs) from a late-onset Alzheimer disease (LOAD) patient and a sex- and age-matched healthy control (CON). For this purpose, peripheral blood mononuclear cells (PBMCs) were first isolated fromblood. These cells were then immortalized with the Epstein-Barr virus to produce a B-lymphoblastoid cell line (B-LCL). The resulting B-LCL was then transduced and reprogrammed into hiPSCs. (**B**) Summary of the clinical evaluation of the LOAD patient and the CON donor. Both were female, of Caucasian origin and over 60 years of age. At the age of 73, the LOAD patient was diagnosed with mild dementia, code F00.1: Dementia of Alzheimer's disease, late onset (type 1) according to the 10^th^ version of the International Statistical Classification of Diseases (ICD-10). She was examined using a Minimal Mental Status Examination (MMSE) and a neuropsychological test battery from the Consortium to Establish a Registry for Alzheimers' Disease (CERAD), including a vocabulary test. She was homozygous for APOE4. The CON subject showed no cognitive impairment. She was homozygous for APOE3. (**C**) Phase contrast images showed the characteristic colony morphology of hiPSCs. Scale bar: 400 µm. Immunofluorescence analyses confirmed the expression of pluripotency markers and their cellular localization in the nucleus. Scale bar: 100 µm. (**D**) The quantification of the immunofluorescent images shown in C using high-content image analysis. Mean ± SEM of n = 3. (**E**) Bisulfite sequencing results of CpG sites in the proximal promoter regions of OCT4 (Bis-2-OCT4, Bis-4-OCT4) and NANOG (Bis-1-NANOG). Top numbers indicate CpG number relative to the transcriptional start sites of OCT4 or NANOG. Each circle corresponds to a single CpG in one bacterial clone for that region. A series of 5 clones are shown. Filled circles represent methylated and empty circles unmethylated cytosines.

# **Figure S2**


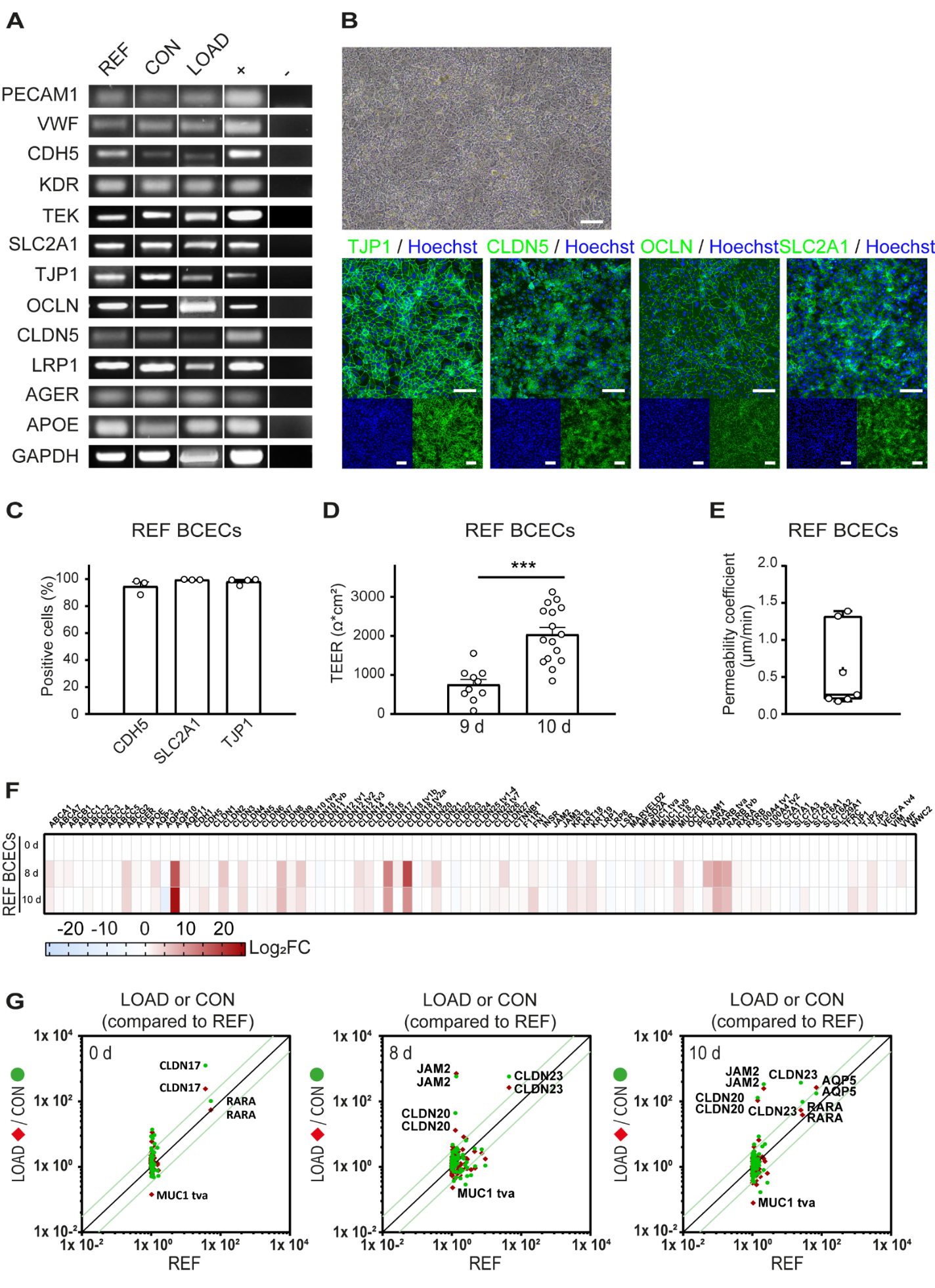


**Characterization of BCECs drived from REF hiPSCs.** Brain capillary endothelial-like cells (BCECs) were obtain from WISCi004-B (REF) human induced pluripotent stem cells (hiPSCs) as refrence for the comparion of MLUi009-A (CON) and MLUi007-J (LOAD). (A) Transcript analysis of endothelial markers in BCECs derived from the WISCi004-B (REF), MLUi009-A (CON) and MLUi007-J (LOAD). Postive (+) and negative (-) samples (no template PCR reactions) are shown as controls (B) REF BCEC’s cobblestone-like morphology at day 10 is shown in a phase contrast image. Immunofluorescent images confirm the expression of BBB marker proteins and their localization to the cell membranes. Scale bar: 100 µm. (C) Almost all REF BCECs were positive in flow cytometry for CDH5, SLC2A1, and TJP1. Mean ± SEM of n = 3 4 independent experiments. (D) Measurement of transendothelial resistance (TEER) across the BCEC layer revealed the highest barrier integrity of REF BCECs at day 10 of differentiation with TEER values > 1000 Ω*cm2. Mean ± SEM of n ≥ 10 independent experiments performed in two different laboratories (HAL and HH). Paired Student's t-test, ***p = 0.0002. (E) REF BCECs showed almost no paracellular transport of sodium fluorescein (NaF), expressed as by the permeability coefficient (PCNaF) at day 10. Box and whisker plot with n = 7 independent experiments performed in two different laboratories (HAL and HH). (F) Comparison of undifferentiated (0 d) REF hiPSCs and differentiated BCECs at day 8 (8 d) and day 10 (10 d) by high-throughput multiplex qPCR analysis of BBB markers (mean of n = 3 independent differentiations). Log2FC for each transcript is plotted on a heatmap as up-regulated (red) and down-regulated (blue) transcripts compared to 0 d. (G) The three digrams show a detailed analysis of the barrier chip for CON and LOAD cells compared to REF cells at day 0 (0 d), day 8 (8 d) and day 10 (10 d). At 0 d iPSCs were compared, while at 8 d progenitor cells and at 10 d BCECs were compared. The scatter plot shows logarithmic values (Log10) for all transcripts in CON (green) compared to REF and LOAD (red) compared to REF along the y-axis relative to REF along the x-axis in a logarithmic scale from 0.01 (1x 10-2) to 10,000 (1x 104) . The black diagonal shows the value at which the CON or LOAD values are identical to the REF values. The green diagonals indicate a 2-fold positive or negative change.

# **Figure S3**


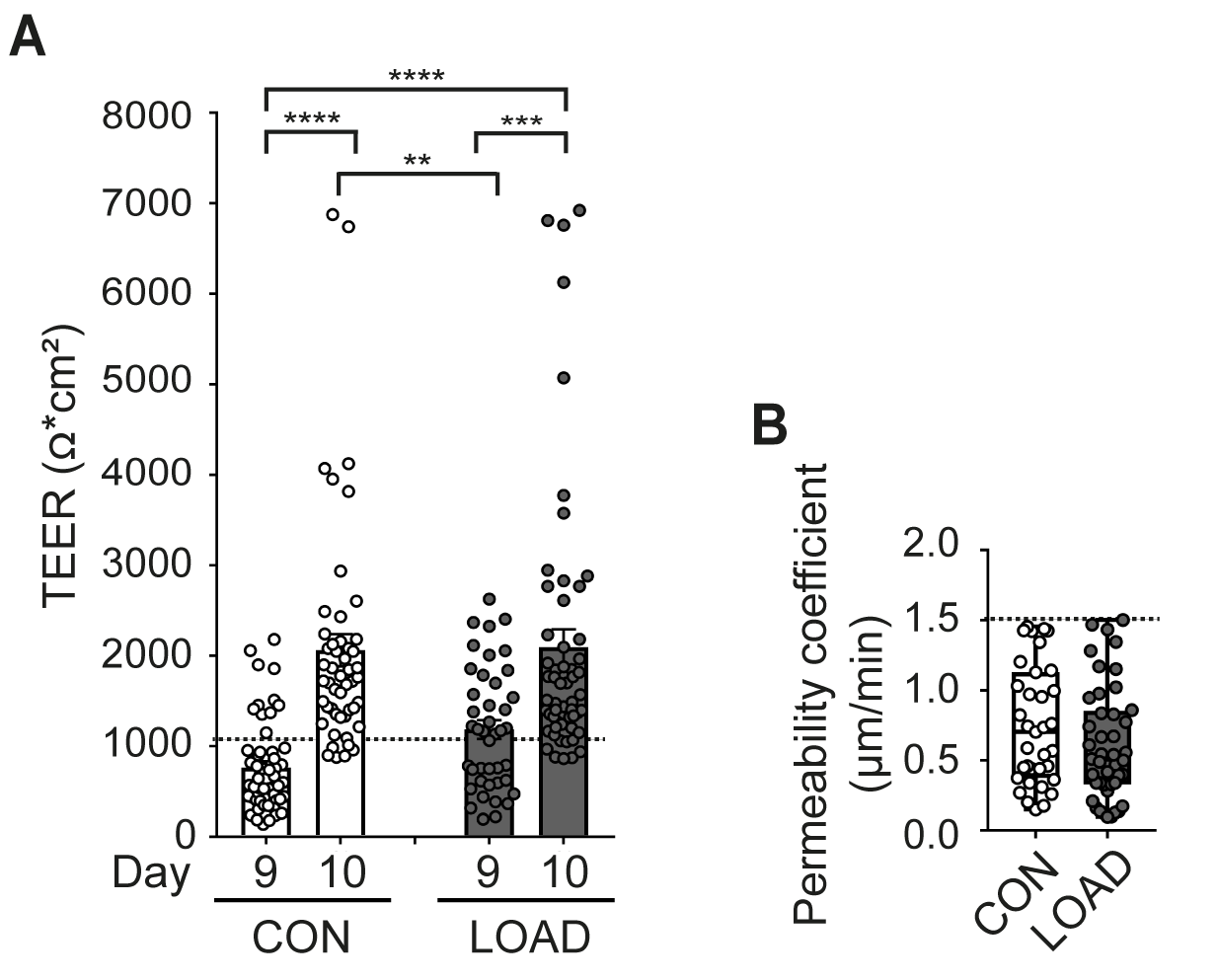


**Interlaboratory comparison - barrier formation and integrity across four indepentent laboratories.** (**A**) Transendothelial electrical resistance (TEER) values measured in the BCEC layer on days 9 and 10 of the differentiation process showed differences in the development of barrier integrity between CON and LOAD models. For all experiments of this study, only inserts with TEER values > 800 Ω*cm^2^ were considered (dotted line). Mean ± SEM of independent experiments (CON n = 53, LOAD n = 57). Two-way ANOVA following *post-hoc* Tukey’s multiple comparison test, *****p* < 0.0001, ****p* = 0.0003, ***p* = 0.0009. (**B**) The permeability coefficient of sodium fluorescein (PC_NaF_) showed a very low permeability and thus hardly any paracellular transport of NaF for CON and LOAD BCECs (PC_NaF_ < 1.5). Box and whisker plots displaying independent experiments (CON n = 35, LOAD n = 44). Unpaired Student’s t-test, *p* = 0,2396.

# **Figure S4**


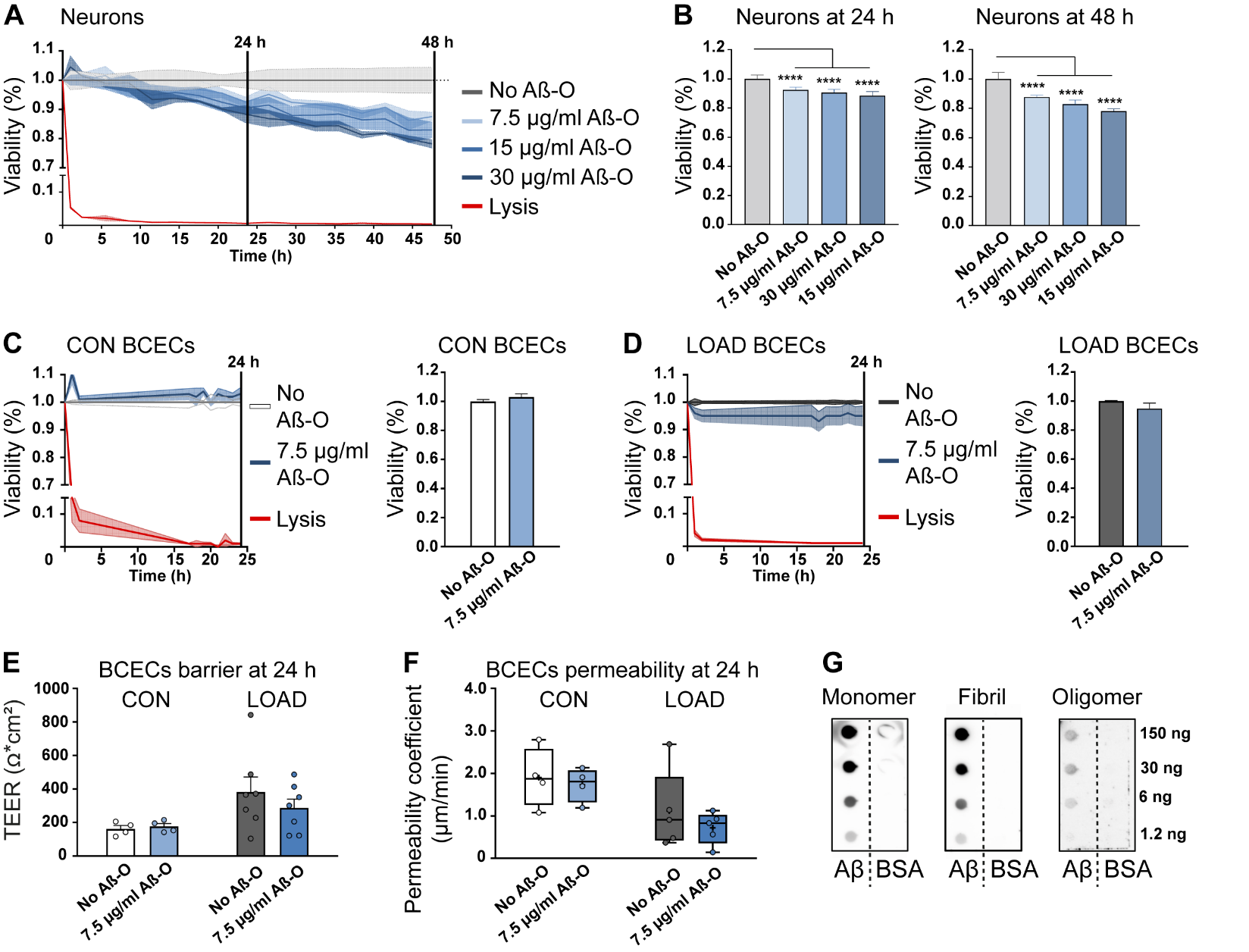


**Effect of amyloid β oligomers on hiPSC-derived neuron and BCEC viability.** (**A**) **Effect of amyloid β oligomers on hiPSC-derived neuron and BCEC viability.** (**A**) As a measure of sensitivity to amyloid-β-oligomers (Aβ-O), cell viability was determined after treatment with Aβ-O in REF neurons because it is this cell type that degenerates in LOAD. The measurement was performed continuously for 48 h with different concentrations, whereby a time- and concentration-dependent effect was observed. (**B**) Viability of REF neurons was significantly decreased in response to Aβ-O treatment after 24 h and 48 h h. Mean ± SD, One-way ANOVA followed by Sidak’s multiple comparison test, *****p* < 0.0001. The viability of CON (**C**) and LOAD (**D**) BCECs was not affected by the treatment with 7.5 µg/ml Aβ-O for 24 h. Mean ± SEM of n = 4 independent experiments. (**E**) Measurement of TEER across the BCEC layer revealed reduced TEER values (TEER < 1000 Ω*cm^2^) indicating reduced barrier integrity of CON and LOAD BCECs at day 11. There were no significant differences between CON and LOAD BCECs or untreated and Aβ-O-treated BCECs. Mean ± SEM of n = 4-7 independent experiments, Paired Student's t-test. (**F**) CON and LOAD BCECs showed increased PC_NaF_ also indicating reduced barrier integrity at day 11. There were no significant differences between CON and LOAD BCECs or untreated and Aβ-O-treated BCECs. Box (median and lower/upper quartile) and whisker (minimum/maximum) plots display n = 4 independent experiments, Paired Student's t-test. (**G**) Dot blot from a filter retardation test showed that besides Aβ-O also Aβ monomers (and peptides) and Aβ fibrils were present in the applied Aβ-O solution (referred to throughout the text as treatment with Aβ-O). Shown are different concentrations of Aβ-O (1.2 ng ‑ 150 ng) compared to the solvent with BSA.

**Table S1**

Gene target list of high-throughput qPCR. Claudin nomenclature according to Mineta et al. [1].

| **Number** | **Symbol** | **Target** | **NCBI RefSeq** |
| --- | --- | --- | --- |
| 1 | ABCA1 | ATP binding cassette subfamily A member 1 | [NM_005502.4](https://www.ncbi.nlm.nih.gov/nuccore/NM_005502.4) |
| 2 | ABCA7 | ATP binding cassette subfamily A member 7 | NM_019112.4 |
| 3 | ABCB1 | ATP binding cassette subfamily B member 1 | NM_000927.4 |
| 4 | ABCC1 | ATP binding cassette subfamily C member 1 | NM_004996.3 |
| 5 | ABCC2 | ATP binding cassette subfamily C member 2 | NM_000392.4 |
| 6 | ABCC3 | ATP binding cassette subfamily C member 3 | NM_003786.3 |
| 7 | ABCC4 | ATP binding cassette subfamily C member 4 | NM_005845.4 |
| 8 | ABCC5 | ATP binding cassette subfamily C member 5 | NM_005688.3 |
| 9 | ABCG2 | ATP binding cassette subfamily G member 2 | NM_004827.2 |
| 10 | ACTB | Actin beta (housekeeping gene) | NM_001101.4 |
| 11 | AGER | Advanced glycosylation end product-specific receptor | NM_001136.5  NM_001206929.2  NM_001206934.2  NM_001206936.2  NM_001206940.2  NM_001206954.2  NM_001206966.2 |
| 12 | APOE | Apolipoprotein E | NM_000041.4  NM_001302688.2  NM_001302689.2  NM_001302690.1  NM_001302691.2 |
| 13 | AQP3 | Aquaporin 3 | NM_004925.4 |
| 14 | AQP5 | Aquaporin 5 | NM_001651.3 |
| 15 | AQP10 | Aquaporin 10 | NM_080429.2 |
| 16 | AQP11 | Aquaporin 11 | NM_173039.2 |
| 17 | B2M | Beta-2-microglobulin (housekeeping gene) | NM_004048.2 |
| 18 | CDH1 | Cadherin 1 | NM_001317186.1  NM_001317185.1  NM_001317184.1  NM_004360.4 |
| 19 | CDH5 | Cadherin 5 | NM_001795.4 |
| 20 | CLDN1 | Claudin 1 | NM_001307.5 |
| 21 | CLDN2 | Claudin 2 | NM_001171095.1 |
| 22 | CLDN3 | Claudin 3 | NM_001306.3 |
| 23 | CLDN4 | Claudin 4 | NM_001305.4 |
| 24 | CLDN5 | Claudin 5 | NM_001130861.1  NM_003277.3 |
| 25 | CLDN6 | Claudin 6 | NM_021195.4 |
| 26 | CLDN7 | Claudin 7 | NM_001307.5 |
| 27 | CLDN8 | Claudin 8 | NM_199328.2 |
| 28 | CLDN9 | Claudin 9 | NM_020982.3 |
| 29 | CLDN10 tva | Claudin 10 | NM_182848.3 |
| 30 | CLDN10 tvb | Claudin 10 | NM_006984.4 |
| 31 | CLDN11 | Claudin 11 | NM_005602.5 |
| 32 | CLDN12 tv1 | Claudin 12 | NM_001185072.2 |
| 33 | CLDN12 tv2 | Claudin 12 | NM_001185073.2 |
| 34 | CLDN12 tv3 | Claudin 12 | NM_012129.4 |
| 35 | CLDN14 | Claudin 14 | NM_012130.3  NM_001146078.2  NM_001146079.1  NM_001146077.1  NM_144492.2 |
| 36 | CLDN15 | Claudin 15 | NM_014343.2  NM_001185080.1 |
| 37 | CLDN16 | Claudin 16 | NM_006580.3 |
| 38 | CLDN17 | Claudin 17 | NM_012131.2 |
| 39 | CLDN18 tv1b | Claudin 18 | NM_016369.3 |
| 40 | CLDN18 tv2a | Claudin 18 | NM_001002026.2 |
| 41 | CLDN19 | Claudin 19 | NM_148960.2 |
| 42 | CLDN20 | Claudin 20 | NM_001001346.3 |
| 43 | CLDN21 | CLDN25, Claudin 21 according to Mineta et al. | NM_001101389.1 |
| 44 | CLDN22 | Claudin 22 | NM_001111319.1 |
| 45 | CLDN23 | Claudin 23 | NM_194284.2 |
| 46 | CLDN24 | Claudin 24 | NM_001185149.1 |
| 47 | CLDN25 tv1-4, tv6 | CLDN1, Claudin 25 according to Mineta et al. | NM_001040181.2  NM_019895.3  NM_001040183.2  NM_001040182.2  NM_001040199.2 |
| 48 | CLDN25 tv7 | CLDN1, Claudin 25 according to Mineta et al. | NM_001040200.2 |
| 49 | CLDN26 | Claudin 26 according to Mineta et al. | NM_001146336.1 |
| 50 | CLDN27 | Claudin 27 according to Mineta et al. | NM_001204210.1  NM_001204211.1  NM_001204212.1 |
| 51 | CTNNB1 | Catenin beta-1 | NM_001098209.1  NM_001904.3  NM_001098210.1 |
| 52 | F11R | F11 receptor, Junctional adhesion molecule A | NM_016946.4 |
| 53 | FN1 | Fibronectin | NM_212474.2  NM_212476.2  NM_212478.2  NM_002026.3  NM_212482.2  NM_001306132.1  NM_001306131.1 |
| 54 | GAPDH | Glyceraldehyde-3-phosphate dehydrogenase (housekeeping gene) | NM_002046.7 |
| 55 | INSR | Insulin receptor | NM_001079817.2  NM_000208.3 |
| 56 | JAM2 | Junctional adhesion molecule 2 | NM_021219.3 |
| 57 | JAM3 | Junctional adhesion molecule 3 | NM_032801.4 |
| 58 | KRT8 | Keratin 8 | NM_001256293.1  NM_001256282.1 |
| 59 | KRT18 | Keratin 18 | NM_199187.1  NM_000224.2 |
| 60 | KRT19 | Keratin 19 | NM_002276.4 |
| 61 | LRP1 | LDL receptor related protein 1 | NM_002332.2 |
| 62 | LRP8 | LDL receptor related protein 8 | NM_004631.4, NM_001018054.2  NM_033300.3  NM_017522.4 |
| 63 | LSR | Lipolysis-stimulated lipoprotein receptor | NM_001260490.2  NM_015925.7  NM_205834.4  NM_205835.4 |
| 64 | MARVELD2 | MARVEL domain containing 2, MARVEL domain-containing protein 2 | NM_001244734.1  NM_001038603.2 |
| 65 | MFSD2A | Major facilitator superfamily domain containing 2A, Sodium-dependent lysophosphatidylcholine symporter 1 | NM_001349821.1  NM_001349823.1 NM_001349822.1  NM_032793.4  NM_001136493.2  NM_001287809.1  NM_0012878 |
| 66 | MUC1 tva | Mucin 1 | NM_001204294.1  NM_001204293.1 NM_001204285.1  NM_001018017.2  NM_001044390.2  NM_00104439 |
| 67 | MUC1 tvb | Mucin 1 | NM_001204296.1  NM_001204297.1  NM_001204295.1  NM_001204292.1  NM_001204291.1  NM_001204289.1 |
| 68 | MUC18 | Mucin 18 | NM_006500.2 |
| 69 | MUC20 | Mucin 20 | NM_152673.3, NM_001291833.1  NM_020790.1  NM_001282506.1 |
| 70 | OCLN | Occludin | NM_001205255.1  NM_001205254.1  NM_002538.3 |
| 71 | PECAM1 | Platelet endothelial cell adhesion molecule 1 | NM_000442.5 |
| 72 | PPIA | Peptidylprolyl isomerase A (housekeeping gene) | NM_021130.4 |
| 73 | RARA | Retinoic acid receptor alpha | NM_000964.4  NM_001024809.4  NM_001145301.3  NM_001145302.3 |
| 74 | RARB tva | Retinoic acid receptor beta | NM_000965.4  NM_016152.3  NM_001290216.2  NM_001290217.1  NM_001290300 |
| 75 | RARB tvb | Retinoic acid receptor beta | NM_000965.4  NM_016152.3  NM_001290277.1 |
| 76 | RXRA | Retinoic acid receptor RXR-alpha | NM_002957.6 |
| 77 | RXRB | Retinoic acid receptor RXR-beta | NM_021976.5 |
| 78 | S100A4 tv1 | S100 calcium binding protein A4 | NM_002961.2 |
| 79 | S100A4 tv2 | S100 calcium binding protein A4 | NM_019554.2 |
| 80 | SLC2A1 | Solute carrier family 2 member 1, Facilitated glucose transporter member 1 | NM_006516.2 |
| 81 | SLC7A1 | Solute carrier family 7 member 1, High affinity cationic amino acid transporter 1 | NM_003045.4 |
| 82 | SLC7A3 | Solute carrier family 7 member 3, Cationic amino acid transporter 3 | NM_032803.5 |
| 83 | SLC7A5 | Solute carrier family 7 member 5, Large neutral amino acids transporter 1 | NM_003486.6 |
| 84 | SLC16A1 | Solute carrier family 16 member 1, Monocarboxylate transporter 1 | NM_003051.3 NM_001166496.1 |
| 85 | SLC16A2 | Solute carrier family 16 member 2, Monocarboxylate transporter 8 | NM_006517.4 |
| 86 | SLC29A1 | Solute carrier family 29 member 1, Equilibrative nucleoside transporter 1 | NM_001078175.2 NM_001078177.1 NM_001304463.1 NM_001304462.1 |
| 87 | TFRC | Transferrin receptor protein 1 | NM_001313966.1  NM_001313965.1 NM_003234.3  NM_001128148.2 |
| 88 | TJP1 | Tight junction protein 1 | NM_003257.4 |
| 89 | TJP2 | Tight junction protein 2 | NM_001170414.2  NM_201629.3  NM_001170416.1  NM_001170415.1  NM_004817.3 |
| 90 | TJP3 | Tight junction protein 3 | NM_001267561.1 |
| 91 | VEGFA | Vascular endothelial growth factor A | NM_001204384.1  NM_001171622.1  NM_001033756.2  NM_001025370.2  NM_001025369.2  NM_001025368 |
| 92 | VIM | Vimentin | NM_003380.4 |
| 93 | VWF | von Willebrand factor | NM_000552.5 |
| 94 | WWC2 | WW-and-C2-domain-containing family of proteins | NM_024949.5 |

**Table S2**

List of primers for transcript analysis

| **Symbol** | **Forward** | **Reverse** | **NCBI RefSeq** | **Reference** |
| --- | --- | --- | --- | --- |
| Bis-2-OCT4 | ttaggaaaatgggtagtagggattt | tacccaaaaaacaaataaattataaaacct | - | [2] |
| Bis-4-OCT4 | ggatgttattaagatgaagatagttgg | cctaaactccccttcaaaatctatt | - | [2] |
| Bis-1-NANOG | agagataggagggtaagtttttttt | actcccacacaaactaacttttattc | - | [3] |
| AGER (also known as RAGE) | gtagattctgcctctgaactc | cttcacagatactcccttctc | NM_001136.5  NM_014795.4  NM_172197.3  NM_001206929.2  NM_001206934.2  NM_001206932.2  NM_001206936.2  NM_001206940.2  NM_001206954.2  NM_001206966.2  NM_001171653.2 | [4] |
| APOE | agacactgtctgagcaggtg | ggggtcagttgttcctccag | NM_000041.3 NM_001302688.1 NM_001302689.1 NM_001302690.1 NM_001302691.1 | - |
| CDH5 | cgcaatagacaaggacataacac | ggtcaaactgcccatacttg | NM_001795.5 | [4] |
| CLDN5 | cggcgactacgacaagaaga | gtcttggaggggaagcgaaa | NM_001130861.1  NM_003277.3 | - |
| GAPDH | caaggtcatccatgacaactttg | gtccaccaccctgttgctgtag | NM_001256799.2  NM_001289746.1  NM_001289745.1  NM_002046.5 | - |
| KDR (also known as VEGFR2) | gtgaccaacatggagtcgtg | ccagagattccatgccactt | NM_002253.4 | - |
| LRP1 | gactacattgaatttgccagcc | tcttgtgggctcggttaatg | NM_002332.3 | [4] |
| OCLN | ccaatgtcgaggagtgggtta | tgccatgggactgtcaactc | NM_001205254.1  NM_001205255.1  NM_002538.3 | - |
| PECAM1 | gagtattactgcacagccttca | aaccactgcaataagtcctttc | NM_000442.5 | [4] |
| SLC2A1 (also known as GLUT1) | tggcatcaacgctgtcttct | agccaatggtggcatacaca | NM_006516.2 | - |
| TEK (also known as TIE2) | ccaaacgtgattgacactgg | tgtgaagcgtctcacaggtc | [NM_000459.5](https://www.ncbi.nlm.nih.gov/entrez/viewer.fcgi?db=nucleotide&id=1798088737)  NM_001290077.2  [NM_001290078.2](https://www.ncbi.nlm.nih.gov/entrez/viewer.fcgi?db=nucleotide&id=2633575831)  [NM_001375476.1](https://www.ncbi.nlm.nih.gov/entrez/viewer.fcgi?db=nucleotide&id=1770499960)  [NM_001375475.1](https://www.ncbi.nlm.nih.gov/entrez/viewer.fcgi?db=nucleotide&id=1770499958) | [4] |
| TJP1 (also known as ZO1) | cggtcctctgagcctgtaag | ggatctacatgcgacgacaa | NM_003257.5  NM_001330239.4  NM_001355014.2  NM_001301025.3  NM_001355012.2  NM_001355013.1 | [5] |
| VWF | cccgaaaggccaggtgta | agcaagcttccggggact | NM_000552.5  [NM_013269.6](https://www.ncbi.nlm.nih.gov/entrez/viewer.fcgi?db=nucleotide&id=1519314891)  [NM_178443.3](https://www.ncbi.nlm.nih.gov/entrez/viewer.fcgi?db=nucleotide&id=1839337252)  [NM_001382362.1](https://www.ncbi.nlm.nih.gov/entrez/viewer.fcgi?db=nucleotide&id=1836650580)  [NM_001197319.3](https://www.ncbi.nlm.nih.gov/entrez/viewer.fcgi?db=nucleotide&id=1676341290)  [NM_001197317.3](https://www.ncbi.nlm.nih.gov/entrez/viewer.fcgi?db=nucleotide&id=1675178798)  [NM_001197318.3](https://www.ncbi.nlm.nih.gov/entrez/viewer.fcgi?db=nucleotide&id=1674985909)  [NM_001004419.5](https://www.ncbi.nlm.nih.gov/entrez/viewer.fcgi?db=nucleotide&id=1674985848) | [4] |

-: not applicable

**Methods additonal file**

**Transcript analysis**

RNA extraction was performed with RNeasy^TM^ Mini Kit (Qiagen) according to the manufacturer’s protocol. 1.0 µg of total RNA were transcribed into cDNA by using Revertaid^TM^ M MuLV RT with buffer, RNase inhibitor, oligo (dT) 18 primers, and deoxyribonucleotide triphosphate (dNTPs; all from Thermo Fisher Scientific). Standard reverse transcriptase PCR (RT-PCR) analysis were performed with 1.0 µl template cDNA in a 25.0 µl reaction, 10x buffer BD, 25 mM MgCl2, 2.5 mM dNTPs, 5.0 U / μl Firepol^TM^ DNA polymerase (all from Solis Biodyne), and 10 pmol / μl of each primer (Biomers). Primers are listed in Table S2.

**Promotor analysis by bisulfite genomic sequencing**

Genomic DNA was isolated from MLUi007-J and MLUi009-A using the DNeasy^TM^ Blood & Tissue Kit and then treated afterwards with the EpiTect^TM^ Fast DNA Bisulfite Kit (both Qiagen) for bisulfite conversion corresponding to manufacturer’s instructions. The bisulfite-modified DNA was cleaned up and then used as a template for PCR to amplify the promoter regions of POU5F1 and NANOG using the HOT Firepol Kit (Solis Biodyne). Primers used for methylation analysis are listed in Table S2. Amplicons were purified with the QIAquick^TM^ Gel Extraction Kit (Qiagen) and ligated into the pGEM^TM^-T Easy Vector System (Promega) overnight at 4°C. Subsequently, JM109 competent cells were transformed with the ligated pGEM^TM^-T Easy constructs and incubated overnight at 37°C. Using the blue-white screening five independent colonies were selected and DNA was extracted with the Monarch^TM^ Plasmid DNA Miniprep Kit (NEB) according to manufacturer’s protocols. Finally, the purified vectors including the promoter sequences were sequenced with M13 primers by Microsynth Seqlab.

**Differences of the *in vitro* BBB model between four independent laboratories**

The cultivation of hiPSC differed between the four independent laboratories. One lab used different hiPSC culture conditions: hiPSC were cultured uner normoxic conditions without gentamycin. 0.5 mM EDTA was used for passaging. For the generation of BCECs, platelet-derived serum was used instead of B-27 and Falcon Cell Culture Inserts were obtained from Corning.

**References additional file**

1. Mineta K, Yamamoto Y, Yamazaki Y, Tanaka H, Tada Y, Saito K, et al. Predicted expansion of the claudin multigene family. FEBS Lett. 2011;585:606–12. doi:10.1016/j.febslet.2011.01.028.

2. Deb-Rinker P, Ly D, Jezierski A, Sikorska M, Walker PR. Sequential DNA methylation of the Nanog and Oct-4 upstream regions in human NT2 cells during neuronal differentiation. J Biol Chem. 2005;280:6257–60. doi:10.1074/jbc.C400479200.

3. Habich A, Szablowska-Gadomska I, Zayat V, Buzanska L, Domańska-Janik K. Epigenetic and molecular signature of human umbilical cord blood-derived neural stem cell (HUCB-NSC) neuronal differentiation. Acta Neurobiol Exp (Wars). 2013;73:143–56. doi:10.55782/ane-2013-1928.

4. Lippmann ES, Azarin SM, Kay JE, Nessler RA, Wilson HK, Al-Ahmad A, et al. Derivation of blood-brain barrier endothelial cells from human pluripotent stem cells. Nat Biotechnol. 2012;30:783–91. doi:10.1038/nbt.2247.

5. Youakim A, Ahdieh M. Interferon-gamma decreases barrier function in T84 cells by reducing ZO-1 levels and disrupting apical actin. Am J Physiol. 1999;276:G1279-88. doi:10.1152/ajpgi.1999.276.5.G1279.
